# Predicting Essential Genes and Proteins Based on Machine Learning and Network Topological Features: A Comprehensive Review

**DOI:** 10.3389/fphys.2016.00075

**Published:** 2016-03-08

**Authors:** Xue Zhang, Marcio Luis Acencio, Ney Lemke

**Affiliations:** ^1^Department of Computer Science, Xiangnan UniversityHunan, China; ^2^Department of Physics and Biophysics, Institute of Biosciences of Botucatu, São Paulo State UniversityBotucatu, Brazil

**Keywords:** essential genes/proteins, machine learning, systems biology, prediction models, network topological features

## Abstract

Essential proteins/genes are indispensable to the survival or reproduction of an organism, and the deletion of such essential proteins will result in lethality or infertility. The identification of essential genes is very important not only for understanding the minimal requirements for survival of an organism, but also for finding human disease genes and new drug targets. Experimental methods for identifying essential genes are costly, time-consuming, and laborious. With the accumulation of sequenced genomes data and high-throughput experimental data, many computational methods for identifying essential proteins are proposed, which are useful complements to experimental methods. In this review, we show the state-of-the-art methods for identifying essential genes and proteins based on machine learning and network topological features, point out the progress and limitations of current methods, and discuss the challenges and directions for further research.

## Introduction

Genome-wide gene deletion studies have shown that a small fraction of genes in a genome are indispensable to the survival or reproduction of an organism (Winzeler et al., 1999; Kamath et al., 2003). These genes are referred as essential genes, and essential proteins are the products of essential genes. The deletion of such essential genes will result in lethality or infertility. Since being essential is highly dependent on the circumstances in which an organism lives, recently systematic attempts have been made to identify those genes that are absolutely required to maintain life, provided that all nutrients are available (Zhang and Lin, 2009). Such experiments have led to the conclusion that the absolutely required number of genes for a bacteria is on the order of about 250–300, which encode proteins to maintain a central metabolism, replicate DNA, translate genes into proteins, maintain a basic cellular structure, and mediate transport processes into and out of the cell.

The identification of essential genes is very important not only for understanding the minimal requirements for survival of an organism, but also for finding human disease genes (Steinmetz et al., 2002) and new drug targets. The genome-wide identification of essential genes is valuable for rational drug design (Lamichhane et al., 2003). Essential proteins in pathogenic organisms can be taken as the potential targets for new antibiotics (Becker and Palsson, 2005); therefore, identifying essential genes in pathogens are very important for the development of antibacterial drugs. There are currently three main types of experimental strategies for the genome-wide discovery of essential genes: gene knockout (Giaever et al., 2002; Chen et al., 2015), gene knockdown (Harborth et al., 2001; Ji et al., 2001; Roemer et al., 2003) and transposon mutagenesis (Gallagher et al., 2007; Langridge et al., 2009). These methods can generate accurate collections of essential genes, but they are expensive, time-consuming and laborious. Furthermore, these experimental methods are not suitable for some complex organisms, especially for humans.

In an attempt to circumvent the above-mentioned experimental constraints, computational approaches capable of accurately predicting essential genes would be of great value. By using such computational approaches, for example, all genes of an organism could be ranked according to their essentiality and, given this list of ranked genes, it would be possible to better guide large-scale experiments for detection of essential genes or facilitate the discovery of drug targets in a way that only the top ranked genes would be tested.

In fact, computational approaches for the prediction of essential genes have already been developed and used for a long time. In 1996, Mushegian and Koonin were the first to develop a computational method to predict essential genes based on comparative genomics (Mushegian and Koonin, 1996). Since then, many computational methods for predicting essential genes and proteins have been developed as a consequence of the accumulation of sequencing data for a large number of organisms as well as collections of experimentally verified essential genes and proteins for some model organisms [currently, the available essential genes and protein databases are DEG (Zhang and Lin, 2009), CEG (Ye et al., 2013), OGEE (Chen et al., 2012), and EGGS (http://www.nmpdr.org/FIG/eggs.cgi)]. These data have enabled researchers to explore the features of essential genes and proteins and, through this exploration, reveal which features are associated with essentiality and, finally, develop computational methods proposed to identify essential genes and proteins.

The features that have been associated with gene and protein essentiality and used to predict essential genes and proteins are based on gene expression, sequence (physicochemical properties of proteins, codon bias, phyletic retention), functional annotation (subcellular location, molecular function, biological process) and network topology (see Table S1 in the Supplementary Material). Among these features, network topological features, i.e., features that numerically represent the arrangement of the elements (nodes and edges) in a network, such as degree centrality (DC; Jeong et al., 2001) and betweenness centrality (BC; Joy et al., 2005; see more details in the Section “Biological Networks and Network Topological Features”), have been popular for the prediction of essential genes and proteins as discussed below.

Since the publication of the paper by Jeong et al. (2001) showing for the first time that there was a positive correlation between a network topological feature, DC, calculated from protein-protein interaction networks (biological networks in which nodes are proteins and edges are the physical interactions between them; see more details in the Section “Biological Networks and Network Topological Features”), and protein essentiality, almost all methods developed to predict essential genes/proteins so far have considered network topology as at least one of the features analyzed. As shown in Table S1 in the Supplementary Material, of the 34 articles collected in a search of the literature for original research articles reporting computational methods for prediction of essential genes and proteins (see details about this procedure in the Supplementary Material), 28 (82%) report the utilization of at least one network topological feature. It is worth to mention that, of these 28 articles, 17 (61%) report the utilization of network topological features either combined (simple combination without any modification of topological feature definition) or integrated (when the own definition of topological feature is modified) with other features.

Another observation in this search of the literature is that of the 28 articles reporting the utilization of network topological features to predict essential genes and proteins, in 12 (43%) the computational methods used were those based on machine learning, a method in which computers make and improve predictions based on some data through learning algorithms (see next sections for details). On the other hand, of the 14 articles reporting the utilization of machine learning for the prediction of essential genes and proteins, 12 (86%) report the utilization of network topological features as learning attributes (features that describe a certain instance; see details in the next section). Therefore, this brief analysis of these 34 papers suggests a strong link between machine learning and network topological features regarding the prediction of essential genes and proteins.

Given the potential importance of this link, we sought in this review to go through the related works reporting the utilization of machine learning and network topological features for the prediction of essential genes and proteins and show the current developments, as well as pointing out the benefits, limitations, and further research directions. To this end, we firstly provide some fundamentals on machine learning, biological networks and network topological features and then present studies on prediction of essential genes and proteins using machine learning and network topological features from three different points of view: from the viewpoint of the biological networks and topological features, from the viewpoint of sources of training and testing data sets, i.e., training and testing in the same or different organisms, and from the viewpoint of machine learning algorithms.

## Fundamentals in machine learning and biological networks

### Machine learning

Machine learning algorithms build models from example inputs in order to make data-driven predictions or decisions. Machine learning tasks are typically classified into three broad categories: supervised learning, unsupervised learning and reinforcement learning, depending on the nature of the learning signal or feedback available to a learning system. Supervised learning aims to infer a function from labeled training data in which each example/instance is a pair consisting of an input object (typically a vector) and a desired output value. The inferred function can be used for mapping new examples. Depending on the outputs, machine learning tasks can be categorized into classification, clustering, regression, density estimation, and dimensionality reduction. For the task of identifying essential proteins/genes, supervised classification algorithms are often used.

In supervised classification, given a set of *N* training examples of the form {(x_1_,y_1_), …, (x_*N*_, y_*N*_)} such that x_*i*_ is the feature vector—set of learning features (or attributes) that are descriptive of the training example (in our case, for example, network topological and sequence features)—of the *i*^*th*^ example and y_*i*_ is its label (in our case, for example, essential and non-essential), a learning algorithm seeks a function *g*: *X*→*Y*, where *X* is the input space and *Y* is the output space. The function *g* is an element of hypothesis space of possible functions *G*. It is sometimes convenient to represent *g* using a scoring function *f: X*×*Y*→*R* such that *g* is defined as returning the *y* value that gives the highest score: *g*(*x*) = *argmax*_*y*_ *f*(*x, y*). Let *F* denote the space of scoring functions. Many learning algorithms are probabilistic models where *g* takes the form of a conditional probability model *g*(*x*) = *P*(*y*|*x*) (e.g., logistic regression), or *f* takes the form of a joint probability model *f* (*x*,*y*) = *P*(*x*,*y*) (e.g., naïve Bayes, and linear discriminant analysis).

There are many ways of measuring classification performance. Accuracy, confusion matrix, log-loss, area under the receiver operating characteristic (ROC) curve (AUC), and precision-recall are all popular metrics. Accuracy simply measures how often the prediction model makes the correct prediction. It is the ratio between the number of correct predictions and the total number of predictions. Although accuracy is simple to calculate, it is unable to make distinction between classes. If the cost of classification for each class is different, or if the test data are imbalanced between classes, we might want to look at how many examples failed for one class vs. another class. In this case accuracy is not enough. A confusion matrix or confusion table shows a more detailed breakdown of correct and incorrect classifications for each class. The rows of the matrix correspond to ground truth labels, and the columns represent the prediction.

A variation of accuracy is the average per-class accuracy, the average of the accuracy for each class. Accuracy is an example of a micro-average, and average per-class accuracy is a macro-average. Log-loss or logarithmic loss can be used if the raw output of the prediction model is a numeric probability. The probability essentially serves as a gauge of confidence. AUC is the area under the ROC curve and shows the sensitivity of the prediction model for plotting the rate of true positives to the rate of false positives. In pattern recognition and information retrieval with binary classification, precision is the fraction of retrieved instances that are relevant, while recall (also known as sensitivity) is the fraction of relevant instances that are retrieved. Precision and recall are often not discussed in isolation, instead either values for one measure are compared for a fixed level at the other measure (precision-recall curve), or both are combined into a single measure, e.g., F-measure.

Various machine learning algorithms have been used for identifying essential genes and proteins. The most commonly used algorithms are support vector machine (SVM) and ensemble learning-based methods (Table 1; for more details on these and other machine learning algorithms, please see Mitchell, 1997). In the Section “Machine Learning Algorithms and Approaches” we further analyze the utilization of these and other algorithms for the prediction of essential genes and proteins.

**Table 1:** Summary of prediction methods using machine learning and network topological features alone or combined with other features^*^.

**Organisms**	**ML algorithms**	**Network type**	**Network features**	**Combined with other features?**	Train/test^**^	**References**
*S. cerevisiae*	NN, SVM	PIN, GCN	DC	No	Same	Chen and Xu, 2005
*S. cerevisiae*	WKNN, SVM, ensemble	PIN	DC	Sequence-related	Same	Saha and Heber, 2006
*S. cerevisiae* *E. coli*	NB	PIN	DC	Sequence-related	Same	Gustafson et al., 2006
*E. coli*	C4.5 decision tree	PIN, TRN, MN	DC	No	Same	Silva et al., 2008
*S. cerevisiae* *E. coli*	SVM	PIN	DC, BC, CC, KL, CCo, EI, CFD	Sequence-related	Same	Hwang et al., 2009
*S. cerevisiae*	Decision tree-based ensemble for prediction; single C4.5 decision tree for description	PIN, TRN, MN	DC, BC, CC, CCo, identicalness	Related to functional annotation	Same	Acencio and Lemke, 2009
*S. cerevisiae*	GEP	PIN	DC, BC, CC, SC, EC, IC, NC, PeC, WDC, ION	Related to functional annotation	Same	Zhong et al., 2013
*P. aeruginosa, E. coli, S. typhinurium*	SVM	MN	RUP, PUP, ND, APL, LSP, NS, NP, NNR, NNNR, CCV, DIR, CP, LS, NDR, NDC, NDRD, NDCD, NDCR, NDCC, NDCRD, NDCCD, BC, CC, EC, eccentricity centrality	No	Different	Plaimas et al., 2010
*E. coli, P. aeruginosa*	Ensemble	GCN	DC, BC	Sequence and gene expression-related	Different	Deng et al., 2011
*E. coli, S. cerevisiae, S. sanguinis, S. pombe*	FWM (NB, logistic regression, genetic algorithm)	PIN	DC, CC, BC, CCo	Sequence and gene expression-related	Different	Cheng et al., 2013
*E. coli, S. enterica, H. influenzae, V. cholerae, P. aeruginosa, Acinetobacter, F. tularensis, H. pylori, C. jejuni, C. crescentus, B. subtilis, S. aureus, S. pneumoniae, S. sanguinis, M. genitalium, M. pulmonis, M. tuberculosis, B. thetaiotaomicron, P. gingivalis, S. cerevisiae, S. pombe*	NB	PIN	DC, CC, BC, CCo	Sequence and gene expression-related	Different	Cheng et al., 2014
*N. crassa, A. fumigatus*	Ensemble	GCN	DC, BC	Sequence and gene expression-related	Different	Lu et al., 2014

* Abbreviations: NN, neural network; WKNN, weighted k-nearest-neighbor; SVM, support vector machine; NB, Naive bayes; GEP, gene expression programming; FWM, feature-based weighted Naïve Bayes model; PIN, protein-protein interaction network; GCN, gene co-expression network; TRN, transcriptional regulatory network; MN, metabolic network; DC, degree centrality; BC, betweenness centrality; CC, closeness centrality; KL, clique level; CCo, clustering coefficient; EI, essentiality index; CFD, common function degree; SC, subgraph centrality; EC, eigenvector centrality; IC, information centrality; NC, edge-clustering coefficient centrality; WDC, weighted degree centrality; RUP, reachable/unreachable products; PUP, percentage of unreachable products; ND, number of deviations; APL, average path length; LSP, length of the shortest path; NS, number of substrates; NP, number of products; NNR, number of neighboring reactions; NNNR, number of neighboring reactions; CCV, clustering coefficient value; DIR, directionality of a reaction; CP, choke point; LS, load score; NDR, number of damaged reactions; NDC, number of damaged compounds; NDRD, number of damaged reactions having no deviations; NDCR, number of damaged choke; NDCC, number of damaged choke point compounds; NDCRD, number of damaged choke point reactions having no deviations; NDCCD, number of damaged choke point compounds having no deviations.

** Same, the sources of training and testing data sets are from same organisms; Different, the sources of training and testing data sets are from different organisms.

### Biological networks and network topological features

A biological network can be represented as weighted or unweighted nondirected or directed graph *Gp*(*P*) = (*Vp, Ep*), where *Vp* = {*p*_*i*_*|p*_*i*_ ∈ *P*} is vertex set that represent biological entities (genes, proteins, enzymes), and *Ep* = {(*p*_*i*_*, p*_*j*_)|for *p*_*i*_*, p*_*j*_ ∈ *P* and *i*≠*j*} is edge set that present their interactions. Let *N* denote the number of nodes in the network and matrix *A* its adjacency matrix whose element *A*_*u, v*_ is 1 if there is an edge between node *u* and node *v*, and 0 otherwise.

There are various types of biological networks, such as protein-protein interaction (PPI) networks (PINs), transcriptional regulatory networks (TRNs), gene co-expression networks (GCNs), metabolic networks (MNs) and integrated networks (integration of PPIs, TRNs and metabolic networks; for a detailed review on the different types of networks, please see Christensen et al., 2007). PINs are the most common used type of biological network (Table 1) for the machine learning-based prediction of essential proteins and genes (see Section “Learning Attributes: Biological Networks and Network Topological Features” for more details).

**Figure 1:**
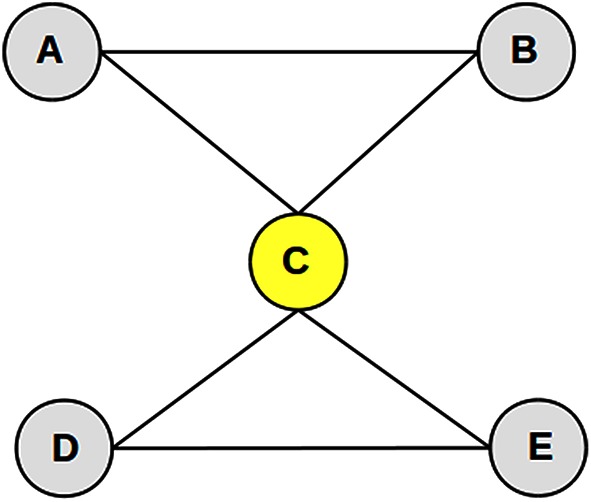
**A toy network showing the calculation of network topological features**. We consider node C (yellow node) as an example to show the calculation of the network topological features. The degree centrality (DC) of node C is 4 because it has 4 edges connecting with nodes A, B, D, and E. The betweenness centrality (BC) of node B is the number of times that node B acts as a bridge along the shortest paths between two other nodes. There are six shortest paths between all other pair of nodes (ACD, ACE, AB, BCD, BCE, DE) of which node C acts a bridge 4 times. Then, BC of node C is 4/6 = 0.66. The closeness centrality (CC) of node C is the reciprocal of the average distance from node C to other nodes. Therefore, CC of node C is 1. The clustering coefficient (CCo) of node C is calculated as the proportion of actual connections among its neighbors (A, B, D, and E) that is, in this case, 2, and the number of all possible connections among its neighbors (in this case, 6). Therefore, CCo of node C is 2/6 = 0.33.

The importance of proteins, genes or enzymes in a biological network is given by the network topological features. These features numerically express the position of the elements (nodes and edges) in a network in relation to all other elements. While there exist many different types of network topological features (Christensen et al., 2007), we will define here those more frequently used for the machine learning-based prediction of essential genes and proteins (Table 1; more details in Section “Learning Attributes: Biological Networks and Network Topological Features”), namely DC, BC, clustering coefficient (CCo) and closeness centrality (CC; Figure 1).

The historically first and conceptually simplest topological feature is degree centrality (DC) that is defined as the total number of edges incident upon a node in the unweighted network (Figure 1), or the sum of weights in the weighted network. It measures the connectivity of a node in the network. Betweenness centrality (BC) of a node is defined as the average fraction of the shortest paths that pass though the node (Figure 1). Closeness centrality (CC; Wuchty and Stadler, 2003) measures how quickly a node communicates with other nodes in the network. The closeness centrality of a node *u* is defined as the reciprocal of the average shortest distances from a node *u* to all other nodes (Figure 1). Local clustering coefficient or clustering coefficient (CCo) is a measure with respect to neighbors' features and is defined as the ratio of the number of edges connecting the neighbors of a node to the maximum number of possible edges among them.

## Studies on prediction of essentiality using machine learning and network topological features

Generally, the development of predictive models in a machine learning approach is accomplished by the following steps: selection of learning attributes (in this case, network topological features), construction of the training and testing data sets (in this case, from the same or different organisms), selection of learning algorithms, design of the machine learning approach and evaluation of the predictive performance of models. We structured this section according to these steps: first we discuss the studies from the perspective of the learning attributes (network topological features), then we discuss the studies from the perspective of the construction of training and testing data sets (in the same or different organisms) and finally we discuss the studies from the perspective of machine learning algorithms and approaches. The discussions are based on the papers shown in Table 1, that is, the 12 papers retrieved by the literature search (see details about this literature search in the Supplementary Material) that report the utilization of network topological features as learning attributes.

### Learning attributes: biological networks and network topological features

As previously mentioned in the Section “Biological Networks and Network Topological Features”, PINs are the most frequently utilized network among all other different types of biological networks from which topological features used as learning attributes are calculated. As shown in Table 1, of the 12 studies reporting the prediction of essential genes and proteins by machine learning and network topological features, nine (75%) report the utilization of PINs to calculate all or part of the network topological networks considered as learning attributes. Why PINs and not MNs or any other network are the most frequently utilized network for the prediction of essential proteins and genes?

We hypothesized that there are two main reasons for which PINs have been the most used biological network for the prediction of essential proteins. The first reason is the pioneering study by Jeong et al. (2001) showing for the first time that there is a positive correlation between a network topological feature calculated from PINs. As mentioned previously, after this demonstration, almost all methods developed to predict essential genes and proteins so far have considered network topology as at least one of the features analyzed. The second reason is the relative abundance of protein-protein interactions data in comparison to other types of interactions. For example, according to the Pathguide database (as of January 2016), there are approximately 260 protein-protein interactions resources, 130 metabolic pathways resources and 80 transcriptional regulatory interactions resources. Therefore, it seems easier to construct a PIN than a MN or a TRN.

A cellular network, however, is not composed only of PPIs. Instead, other interactions are intertwined with PPIs, i.e., metabolic and transcriptional regulatory interactions. Although PINs may provide a global view of cellular signaling, essential genes and proteins are also involved in metabolic processes and direct regulation of gene transcription, that is, essential genes can be present only in non-PINs (MNs or TRNs). Hence, by using only PPIs, researchers are unable to predict essential genes in MNs or TRNs. However, in some cases (specifically six studies as shown in Table 1), biological networks other than PINs were used to predict essential genes and proteins in a machine learning approach as described below.

While Plaimas et al. (2010) used MNs—bipartite graphs in which vertices (nodes) are reactions and enzymes and edges are metabolites—to identify essential enzymes to find potential drug targets in pathogens, Silva et al. (2008) and Acencio and Lemke (2009), in an attempt to predict essential genes considering the whole cellular network, combined a PIN, a MN and a TRN in an integrated network. This integrated network is a unweighted graph in which two genes, g_1_ and g_2_, coding for proteins p_1_ and p_2_, are interacting pairs if p_1_ and p_2_ interact physically or the transcription factor p_1_ directly regulates the transcription of gene g_2_ (p_1_ binds to the promoter region of g_2_) or the enzymes p_1_ and p_2_ share metabolites. Moreover, other authors (Chen and Xu, 2005; Deng et al., 2011; Lu et al., 2014) used GCNs to predict essential genes. Although the interactions in GCNs are functional (usually they are Pearson correlation values between gene expression profiles above a given threshold) instead of physical interactions, co-expressed genes are often involved in the same pathway or similar cellular function, and interacting proteins are frequently co-expressed (Jansen et al., 2002). Thus, these functional interactions induced by the correlation among gene expression profiles might influence gene essentiality.

From this variety of biological networks, practically the same types of network topological features are used as learning attributes to predict essential genes and proteins. As previously mentioned, the most frequently used network topological features are DC, BC, CCo, and CC. Of the 12 studies in Table 1, DC was used in all studies (100%), BC in eight studies (67%), CCo in four studies (33%) and CC in six studies (50%). In addition, nine (75%) studies report the utilization of network topological features in conjunction with other features (Table 1). The utilization of these network topological features, either alone or in conjunction with other features, in machine learning approaches is justified by some previous demonstration of their relationships with essentiality, at least in PINs of *S. cerevisiae*. While Jeong et al. (2001), as already discussed here, revealed a positive correlation between DC and protein essentiality, Joy et al. (2005) found that proteins with high BC are more likely to be essential. Regarding CCo and CC, Yu et al. (2007) and Hwang et al. (2009) showed that essential proteins tend to have higher values of CCo and CC than non-essential proteins, respectively.

Other types of network topological features were also used as learning attributes to predict essential genes and proteins. These include other “pure” network topological features, i.e., the topological feature is calculated directly from the network structure without further modification, and integrated network topological features, i.e., the topological feature derived from features directly calculated from the network structure but modified according to the other biological or network features.

Included in the group of the other pure network topological features used to predict essential genes and proteins is the subgraph centrality (SC; Estrada and Rodríuez-Veláquez, 2005). For a node *u*, SC(*u*) counts the total number of closed walks in which a given protein/gene takes part in the network and gives more weights to closed walks of short lengths. Closed walks are related to the network subgraph, thus SC accounts for the number of subgraphs in which a protein/gene participates, giving more weights to smaller subgraphs. Eigenvector centrality (EC) is also a pure network topological features that measures the influence of a node in a network. It assigns relative scores to all nodes in the network based on the concept that connections to high-scoring nodes contribute more to the score of the node in question than equal connection to low-scoring nodes. EC is defined as the principal eigenvector of the adjacency matrix *A* defining the network (Bonacich, 1987). In biological networks, a protein or gene is considered central if it has a high eigenvector score, which means that it is adjacent to other proteins/genes that themselves have high scores. Finally, another two pure network topological features already used for the prediction of essential genes and proteins are the information centrality (IC) (Stephenson and Zelen, 1989), defined as harmonic mean lengths of paths ending at the node, and eccentricity centrality that is the maximum distance from a given node to all other nodes in a graph. Additional pure network topological features specific for a given type of network is discussed later.

Also, in this group of pure network topological features, we can find some features specially created to predict essential genes and proteins. Hwang et al. (2009), for example, created the clique level (KL), defined as the size of largest clique containing the node and the essentiality index (EI), that is the proportion of essential proteins interacting with a given node. Wang et al. (2012) created the edge-clustering coefficient centrality (NC), defined as the sum of the edge-clustering coefficient of the edges belonging to a node. The NC can be seen as the weight for an edge to capture the degree of modularity that the edge involves in, which is defined as the number of triangles to which a given edge belongs, divided by the number of triangles that might potentially include it. There are other pure network topological features created especially, at least a priori, for the prediction of essential genes and proteins that deserve special attention since they are calculated from MNs and integrated networks. These are discussed below.

Plaimas et al. (2010) reported the use of combining features derived from MNs with gene sequence and expression data to identify essential proteins. They utilized regular topological features from the MN—DC, BC, EC and eccentricity—and in addition created four novel topological feature categories. These new categories were “deviation,” “local topology,” “choke points and load scores,” and “damage.” Of these new categories, all the features relating to “deviation” and “local topography,” and most of the features related to “damage,” were specially created for the prediction of essential enzymes.

Silva et al. (2008) and Acencio and Lemke (2009), on the other hand, created topological features derived from the integrated network (integration of PIN, MN, and TRN) to predict essential genes. These features, except identicalness, are all derived from DC and BC and include degree centralities (indegree and outdegree for transcriptional regulatory and metabolic interactions) and betweenness centralities for each type of interaction. Identicalness is the number of genes with identical network topological characteristics).

Besides the above-mentioned pure network topological features, network topological features belonging to the group of the integrated features were also specifically created to predict essential genes and proteins by machine learning approaches. These features are PeC (Li et al., 2012), weighted degree centrality (WDC; Tang et al., 2012) and ION (Peng et al., 2012). PeC captures the co-clustering and co-expression properties of essential proteins by using Pearson correlation coefficient of gene expressions and edge clustering coefficient. WDC (Tang et al., 2012) also integrates network topology with gene expression profiles. ION (Peng et al., 2012) integrates the orthology with PPI networks using an iteration strategy, which is based on the assumption that essential proteins are more evolutionarily conserved and frequently bind each other.

The utilization of these different combinations of biological networks and network topological features associated or not with sequence, gene expression and functional annotation-related features for the prediction of essential genes by machine learning approaches is a clear indication that researchers have made great efforts to improve the prediction models of essentiality since the study by Chen and Xu (2005). For this purpose, different combination of learning attributes and machine learning algorithms have been tested as shown above in this Section. Therefore, of all models developed in the 12 studies shown in Table 1, which should be considered the best model to predict essential genes and proteins?

It is difficult to answer this question since the direct comparison of the prediction performances of the models developed in these 12 studies is impractical. Taking in consideration that we are interested in the predictability of essentiality by network topological features, then, for a reliable comparison among models, it would be necessary that all models were based on the same values of network topological features, i.e., based on the same type of biological networks. As can be observed in Table 1, different biological networks were used. But it is important to mention that “same type of biological network” does not mean simply that all networks are PINs or MNs. Besides that, all networks should be constructed from interactions collected from the same version of the same database. There exist many different databases of a certain type of interaction (e.g., DIP, BioGRID and IntAct for PPIs) and, usually, these databases are regularly updated. Different databases or newer versions of a given database will have different sets of interactions that, in turn, will give rise to new networks with distinct structures and, consequently, different values of network topological features. As an example, we can cite the studies by Hwang et al. (2009) and Acencio and Lemke (2009). In both studies, PINs of *Saccharomyces cerevisiae* were created; however, the interactions of the PIN constructed in the study by Hwang et al. (2009) were collected from the version ScereCR20070107 of DIP and the interactions of the PIN constructed in the study by Acencio and Lemke (2009) were gathered from the version 2.0.42 of the BioGRID database. Therefore, the prediction performances of the models created by these authors cannot be reliably compared. As an additional example we can cite the studies by Saha and Heber (2006) and Gustafson et al. (2006). Although in both studies the PPIs were gathered from DIP, these authors did not show the versions from which PPIs were taken. So, the models produced in these studies cannot also be reliably compared.

Despite the heterogeneity shown above, what we can learn from the analysis of these 12 studies is that integrating other types of biological features (sequence, gene expression, and functional annotation-related features) to network topological features improves the prediction of essential genes and proteins. As an example, we cite the studies by Hwang et al. (2009) and Acencio and Lemke (2009). Using only network topological features as learning attributes, Hwang and co-workers achieved a F-measure of 0.68 and Acencio and Lemke achieved an AUC of 0.77. When these authors combined network features with sequence-related features (in the case of Hwang and co-workers) or functional annotation-related features (in the case of Acencio and Lemke), they achieved, respectively, a F-measure of 0.73 and an AUC of 0.80.

Thus, it seems that only network topological features are not enough to distinguish essential from non-essential genes and proteins. This raises the following question: is the positive correlation between essentiality and network topological features only an artifact of a possible bias (essential genes and proteins are the focus of more studies and therefore tend to have higher values of network topological) present in the networks mainly derived from small scale experiments? If so, this could explain the moderate predictability of essentiality by network topological features. While Coulomb et al. (2005) showed that gene essentiality seems largely unrelated to many network topological features, Zotenko et al. (2008) showed that high connected proteins are essential due to their involvement in essential complex biological modules, a group of densely connected proteins with shared biological function that are enriched in essential proteins. Regardless the resolution of this debate, a large-scale study for evaluating how well essential genes and proteins can be predicted solely by network topological features is necessary to confirm this moderate prediction performance.

### Sources of training and testing data sets (same or different organisms)

Prediction methods for essential genes and proteins often use supervised classification methods to build a model based on a variety of features related to gene and protein essentiality. Usually, most of the known essential and nonessential genes and proteins in an organism are used as training data, and some genes and proteins are left out as testing data. Cross-validation has often been used to evaluate the model. In this type of method, prediction models can only be constructed based on those organisms that most of their essential genes and proteins are experimentally determined.

However, to predict essential genes and proteins across related organisms, especially distantly related organisms, would be more useful since it can enable us to quickly find out the essential genes and proteins for unstudied organisms. Until now, only relatively few researches have been used machine learning to predict essential genes and proteins across related organisms in comparison with those using machine learning to predict essential genes and proteins within an organism. And often, homology mapping has been used to predict essential genes in newly sequenced organisms taking in consideration known essential genes and proteins (Holman et al., 2009; Zhang and Lin, 2009; Juhas et al., 2012). Homology mapping-based methods assume that those genes that are homologous to known essential genes are likely to be essential. These methods can only find out essential genes with conserved orthologs among species that often account for a small portion of target genome. In addition, a number of conserved genes are nonessential and some essential genes have no orthologs in reference organisms.

Some researchers recently have focused on predicting essential proteins across related organisms by using machine learning. Sequence features have been often used to train learning algorithms in some reference organisms and then the created models have been used to predict essential genes in other related organisms (Mushegian and Koonin, 1996; Seringhaus et al., 2006). As these types of methods are not the focus of this review, we only focus on prediction methods using both machine learning and network topological features alone or in combination with other features.

The first prediction method using both machine learning and network topological features to predict essential genes across related organisms was that developed by Deng et al. (2011). These authors used as learning attributes not only network topological features, but they also used gene expression-related features and sequence features. As network topological features, authors considered DC and BC derived from gene co-expression networks. The final prediction model was constructed from *E. coli* data and then applied to predict essential genes in *Pseudomonas aeruginosa PAO1* and *Bacillus subtilis*. These cross-organism predictions yielded AUC scores between 0.69 and 0.89.

Using exactly the same strategy developed by Deng et al. (2011) and Lu et al. (2014) developed a model to predict a compendium of essential genes in *Aspergillus fumigatus* by transferring known essential gene data from another filamentous fungus, *Neurospora crassa*. The constructed model predicted 1674 essential genes in *A. fumigatus* and, by conducting knockout experiments to verify the predictions, they validated the accuracy and wide applicability of the machine learning approach.

Plaimas et al. (2010) sought to identify essential genes across organisms by training the learning algorithm with *Pseudomonas aeruginosa* and testing it with *E. coli*, and vice versa. They used a broad variety of topological features derived from metabolic network, sequence characteristics and co-expression properties potentially associated with essentiality, such as flux deviations, centrality, codon frequencies of the sequences, co-regulation and phyletic retention. Using cross-validation, the authors evaluated the prediction performance of the models applied to the related species and they found that these models achieved reliable results with AUC varying from 0.75 to 0.81.

Cheng et al. (2013) developed a novel machine learning-based approach called feature-based weighted Naïve Bayes model (FWM) to predict essential genes (see more details about this method in the Section “Machine Learning Algorithms and Approaches”). Using the combination of network topological and sequence features as learning attributes, Cheng et al. (2013) showed that FWM can reliably identify essential genes between different species: the AUC values ranged from 0.77 for *Streptococcus sanguinis* as the training species and *Schizosaccharomyces pombe* as the testing species to 0.95 for *E. coli* as the training species and *S. sanguinis* as the testing species.

Cheng et al. (2014) constructed a cross-organism prediction model based on 15 features derived from domain properties, PINs, genomic sequence, homology properties, phyletic gene age, and gene expression. Network topological features include DC, CC, BC, and CCo. The model was applied reciprocally to predict essential genes in 21 microorganisms. Taking in consideration the AUC values of the different pairs of training and test sets, the authors claimed four criteria for training set selection for predicting essential genes across related organisms: (1) essential genes in training set should be reliable; (2) the growth conditions in which essential genes are defined should be consistent in training and prediction sets; (3) species used as training set should be closely related to the target organism; (4) organisms used as training and prediction sets should exhibit similar phenotypes or lifestyles. Considering the assumption that in supervised learning the training and testing data are drawn from the same distribution, these four criteria are straightforward.

### Machine learning algorithms and approaches

As previously mentioned, the most commonly used machine learning algorithms are ensemble learning-based methods and SVM (Table 1). Of the 12 studies reporting the prediction of essential genes and proteins by machine learning and network topological features, four (33%) studies report the utilization of ensemble algorithms and four (33%) studies report the utilization of SVM. The remaining algorithms are naïve Bayes (NB), neural network (NN), weighted k-nearest neighbors (WKNN), C4.5 decision tree, gene expression programming (GEP), logistic regression and genetic algorithm. We will further discuss the utilization of the C4.5 decision tree and ensemble learning-based methods. These two algorithms deserve special attention as shown below.

The utilization of C4.5 decision tree for the prediction of essential genes deserves to be discussed because, beyond predicting, this algorithm can also be used for knowledge acquisition to describe patterns in data sets. Decision trees are decision support tools inferred from the training data that use a graph of conditions and their possible consequences. The structure of a decision tree consists of a root node representing the most important condition for discriminating classes, internal nodes representing additional conditions for class discrimination under the main condition, and leaf nodes representing the final classification. So, one can learn the conditions for classifying instances in a given class by following the path from the root node to the leaf node (Kingsford and Salzberg, 2008). Silva et al. (2008) and Acencio and Lemke (2009) used decision trees as human-readable descriptors from which conditions under which genes tend to be essential could be found. Through the analysis of C4.5 decision trees, Silva et al. (2008) showed that the main factors to define essentiality in *E. coli* was the protein physical interaction and the number of metabolic interactions and Acencio and Lemke (2009) showed that the most important factors determining gene essentiality in yeast are the number of protein physical interactions, the nuclear localization of proteins and the number of regulating transcription factors.

While decision trees are powerful learning algorithms in the sense that they provide a human-readable structure that describe the conditions for which characteristics of interest (classes) can be distinguished by certain learning features, ensemble learning algorithms—combination of multiple learning algorithms—are also powerful learning algorithms but, in their case, the prediction power is related to the improvement of the prediction performance in comparison to individual algorithms. Many studies have showed that by using ensemble algorithms is possible to obtain better predictive performance than could be obtained from any of the constituent individual learning algorithms (Leblanc and Tibshirane, 1996; Opitz and Maclin, 1999; Polikar, 2006). Regarding specifically the prediction of essential genes and proteins, have the ensemble algorithms been effective in improving the prediction? This is a question that is not possible to be currently answered since none of the studies that have used ensemble learning, except for that by Saha and Heber (2006), have also showed the prediction performances of the individual algorithms embedded in the ensemble. Although Saha and Heber (2006) have shown the performances of both individual algorithms and the ensemble algorithm, it remains unclear if an ensemble is really effective in improving the prediction of essential proteins because, in this study, only one of the evaluated performance metrics seemed to be improved, the recall. For individual algorithms (SVM and KNN), the values of recall were 0.74 and 0.77 and for the ensemble, the value was 0.81. On the other hand, AUC values for individual and ensemble algorithms were virtually the same (SVM, 0.82; WKNN, 0.81; ensemble, 0.82). Therefore, it would be necessary a large comparison study to clearly show the advantages of using ensemble algorithms over individual learning algorithms in the prediction of essentiality.

Still regarding comparison among machine learning algorithms, it would be also interesting to discuss the comparison of the prediction performances among individual learning algorithms applied to the same training and testing set. This is useful since each type of machine learning algorithm is adapted to a certain type of data (Mena, 1999) and, therefore, testing different algorithms seems a good practice to find the algorithm with the best performance to a given training and testing set. Of the 12 studies analyzed here, only one study reports the values of prediction performance for more than one individual machine learning algorithms, namely the study by Saha and Heber (2006). As previously discussed, these authors show the values of prediction performances of SVM and WKNN when applied to a same training and testing set. Therefore, it seems that the exercise of comparing learning algorithms considering the same training and testing set is unusual despite this importance. However, we can hypothesize that researchers do compare the results of many different algorithms applied to the same training and testing set, but this is likely to be done only in-house. They probably report only the algorithms showing the highest values of prediction performance.

Finally, it is important to discuss the impact of the correlation among the network topological features on the prediction performance of models created by training of the machine learning algorithms. Some network topological features have shown to be highly correlated, such as DC and BC, for example (Valente et al., 2008; Sinha and Nagarajaram, 2014; Ronqui and Travieso, 2015), and these network topological features have been often used together as learning attributes to predict essential genes and proteins (Table 1). For machine learning algorithms, the concomitant utilization of highly correlated features, i.e., phenomenon of multicollinearity among features, in the set of learning attributes does not only degrade the performance of some prediction models, but it also increases the complexity without necessarily increasing the prediction performance of the created model. Ideally, therefore, only one feature of a pair or a set of highly correlated features should be kept in a machine learning approach. However, of the 12 analyzed studies, only one study (Cheng et al., 2013) reports the assessment of the correlations among network topological features and the development of a method called feature-based weighted Naïve Bayes model (FWM) to overcome the phenomenon of multicollinearity among features. Moreover, it is important to observe that in all studies in which more than one network topological feature were used as learning attributes (Table 1), DC and BC, which have been consistently shown to be highly correlated (Valente et al., 2008; Sinha and Nagarajaram, 2014; Ronqui and Travieso, 2015), were used together. So, the prediction performances of the models created in these studies should be carefully considered as preferably either DC or BC should have been used as one of the learning features.

## Discussion

The importance of identifying essential genes and the limitations of experimental methods has stimulated researchers to predict essential genes *in silico*. The accumulation of various omics data for a large number of organisms and the collections of essential genes for some model organisms have enabled researchers to explore the characteristics of essential genes and the factors that make essential genes different from nonessential genes. Many computational methods have been proposed to predict essential genes, and at the same time, many features have been found to be related with gene essentiality. In this review, we showed some recent progress in predicting essential genes by using machine learning and network topological features. In the following, we will discuss some main challenges in this research field.

Most studies using network topological features using machine learning have focused on very few model organisms, for example, *S. cerevisiae* and *E. coli*, due to the relatively more complete interactome data for these organisms. It may be more useful to construct reliable interacting networks for more organisms by using the currently proposed methods of predicting protein-protein interactions (for a review on this subject, please see Rao et al., 2014). By doing this, the studies for identifying essential proteins can be conducted in more organisms and, consequently, the correlation between the proposed features and gene essentiality can be evaluated in more organisms.

In addition, while many features have been found to be associated with gene essentiality, both correlation level and predictive power may vary in different organisms. For example, protein length is correlated with gene essentiality in both *S. cerevisiae* and *E. coli;* however, small proteins in *E. coli* tend to be essential while small proteins tend to be nonessential in *S. cerevisiae.* Investigating the relationship between the variation of correlation level and predictive power for gene essentiality of features and the evolution direction of organisms should be more useful and interesting. Prediction methods are expected to be more powerful if they consider such relationship. At the same time, few studies have been performed to investigate whether network features also show similar variation across different organisms. If not, network features would be superior to those derived from sequence data for building useful models to predict essential genes and proteins across distantly related organisms.

The currently proposed machine learning-based prediction methods often utilize supervised learning. In supervised learning, the training and testing data are assumed to be drawn from the same distribution. So it is suitable for them to be used for the task of training and testing in the same organisms. However, predicting essential genes across related organisms are more interesting and practical. As the correlation between features and essentiality and the prediction power of these features for discriminating essential from non-essential genes and proteins vary in different organisms, the training data and the testing data are likely to have different distributions for the task of predicting essential genes across organisms. The difference in general increases with the evolutionary distance of the training and testing organisms (Cheng et al., 2014). So it is expected that supervised classification methods have low predictive power for distantly related organisms. Regard to the difference of feature distribution in different organisms, new prediction frameworks should be studied to only transfer useful knowledge from training organisms to testing organisms. In this regard, transfer learning (Pan and Yang, 2010) may provide a useful learning framework for the task of predicting essential genes across distantly related organisms.

To conclude, despite the great progress that has been made in the machine learning-based prediction of essential genes using network topological features since the publication of the pioneering study in the field (Chen and Xu, 2005; Table 1), it is worth to mention that all these models are predictive of “constitutive” essential genes, that is, genes that are essential regardless the growth condition. However, many genes considered as essential under certain growth condition might not play as critical role in another condition (Tong et al., 2004; Nichols et al., 2011). These are the so-called conditionally essential genes, i.e., nonessential genes that become essential depending on the environment conditions. The development of machine learning approaches for the prediction of these conditionally essential genes using network topological features will be the main challenge in the near future considering that the network topological characteristics of these genes remain unexplored.

## Author contributions

XZ, MLA, and NL drafted, revised, and finalized the manuscript.

### Conflict of interest statement

The authors declare that the research was conducted in the absence of any commercial or financial relationships that could be construed as a potential conflict of interest.
