# Corrigendum: Predicting Essential Genes and Proteins Based on Machine Learning and Network Topological Features: A Comprehensive Review

**DOI:** 10.3389/fphys.2016.00617

**Published:** 2016-12-06

**Authors:** Xue Zhang, Marcio L. Acencio, Ney Lemke

**Affiliations:** ^1^Department of Computer Science, Xiangnan UniversityHunan, China; ^2^Department of Physics and Biophysics, Institute of Biosciences of Botucatu, São Paulo State UniversityBotucatu, Brazil

**Keywords:** essential genes/proteins, machine learning, systems biology, prediction models, network topological features

In the original article, the author Xue Zhang's affiliation is “*Department of Computer Science, Xiangnan University, Hunan, China”*. Because the author changed her affiliation to “School of Computer and Software Engineering, Huaiyin Institute of Technology, Huai'an, Jiangsu, China” and the publishing fees will be paid by the new affiliation, this has been added as a present address.

The original article has been updated.

## Conflict of interest statement

The authors declare that the research was conducted in the absence of any commercial or financial relationships that could be construed as a potential conflict of interest.

