# Supplementary material for: Predicting Essential Genes and Proteins Based on Machine Learning and Network Topological Features: A Comprehensive Review
Source: Front Physiol. 2016 Mar 8;7:75. doi: 10.3389/fphys.2016.00075 (PMC4781880; doi:10.3389/fphys.2016.00075)
Supplement: Supplementary file 1 [file DataSheet1.PDF]

*Supplementary Material*

# Predicting essential genes and proteins based on machine learning and network topological features: a comprehensive review

**Xue Zhang*, Marcio Luis Acencio*, Ney Lemke**

**Correspondence:** Corresponding Authors: lindajia03@gmail.com (Xue Zhang) and mlacencio@ibb.unesp.br (Marcio Luis Acencio)

*These authors contributed equally to this work.

## 1 Searching for articles describing the prediction of essential genes/proteins

The search of the literature for original research articles reporting computational methods for prediction of essential genes and proteins as mentioned in the section "Introduction" in the main manuscript was performed as follows:

a. Selection of the advanced search tool of the Web of Science as the search tool;

b. Utilization of the following query to retrieve the articles reporting the computational prediction of essential genes and/or proteins. We selected expressions and terms most likely to be related to the prediction of essential genes using computational methods:

**TI=("predicting essential genes" OR "predicting essential proteins" OR "prediction of essential genes" OR "prediction of essential proteins" OR "identification of essential genes" OR "identification of essential proteins" OR "identifying essential genes" OR "identifying essential proteins" OR "predictability of essential genes" OR "predictability of essential proteins" OR "prediction of gene essentiality" OR "prediction of protein essentiality" OR "predicting gene essentiality" OR "predicting protein essentiality" OR "identification of gene essentiality" OR "identification of protein essentiality" OR "identifying gene essentiality" OR "identifying protein essentiality") AND TS=("computational" OR "in silico")**

c. This query resulted in 38 articles. The original (raw) result taken from the Web of Search is shown in the section "List of the 38 retrieved articles" at the end of this Supplementary Material.

d. Manual curation of the 38 retrieved articles to exclude reviews and original articles reporting experimental prediction and identification of essential genes and/or proteins. Through this procedure, we collected 30 articles (Table S1). Moreover, we extracted from these articles the type of attributes used in the prediction (network topology, sequence, gene expression, functional annotation) and the

type of computational strategy employed in this task (ranking-based methods and machine learning-based approaches).

e. Verification of the citations of the 30 articles collected in the step "c" leading to potential articles describing the computational prediction of essential genes/proteins. Through this procedure, we retrieved more four articles (Table S1).

## 2 Supplementary Table

Table S1. Summary of the 34 articles reporting the prediction of essential genes and proteins found by a manual search of the scientific literature as described above. Articles are presented in chronological order.

| Article (Reference) | Features | Computational Method |
|---|---|---|
| Jeong et al. (2003) | Gene expression, network topology (combination**) | Ranking-based method |
| *Chen and Xu (2005) | Sequence, gene expression, network topology (combination) | Machine learning |
| Estrada (2006) | Network topology | Ranking-based method |
| *Saha and Heber (2006) | Sequence, network topology (combination) | Machine learning |
| Gustafson et al. (2006) | Sequence, network topology (combination) | Machine learning |
| Seringhaus et al. (2006) | Sequence | Machine learning |
| Silva et al. (2008) | Network topology | Machine learning |
| Acencio and Lemke (2009) | Network topology, functional annotation (combination) | Machine learning |
| Hwang et al. (2009) | Sequence, network topology (combination) | Machine learning |
| Holman et al. (2009) | Sequence | Ranking-based method |
| Plaimas et al. (2010) | Sequence, gene expression, network topology (combination) | Machine learning |
| Deng et al. (2011) | Sequence, gene expression, network topology (combination) | Machine Learning |

| | | |
|---|---|---|
| *Liu et al. (2011) | Sequence | Ranking-based method |
| Ren et al. (2011) | Network topology | Ranking-based method |
| Juhas et al. (2012) | Sequence | Ranking-based method |
| Peng et al (2012) | Sequence, network topology (integration***) | Ranking-based method |
| Li et al. (2013) | Network topology (integration) | Ranking-based method |
| Luo and Ma (2013) | Network topology (integration) | Ranking-based method |
| Lu et al.(2013) | Network topology | Ranking-based method |
| Sarangi et al. (2013) | Sequence | Machine Learning |
| Zhong et al (2013) | Network topology, subcellular localization (combination) | Machine Learning |
| Cheng et al. (2013) | Network topology, sequence, gene expression (combination) | Machine Learning |
| *Song et al. (2014) | Sequence | Ranking-based method |
| Cheng et al. (2014) | Network topology, sequence, gene expression (combination) | Machine Learning |
| Tang et al. (2014) | Network topology, gene expression (integration) | Ranking-based method |
| Lu et al. (2014) | Sequence, gene expression, network topology (combination) | Machine Learning |
| Luo and Zhang (2014) | Network topology, Gene Ontology (integration) | Ranking-based method |
| Luo and Kuang (2014) | Network topology | Ranking-based method |
| Wang et al. (2014) | Network topology, gene expression | Ranking-based method |
| Zhao et al. (2014) | Network topology | Ranking-based method |
| Li et al. (2015) | Network topology (integration) | Ranking-based method |

| | | |
|---|---|---|
| Luo and Qi (2015) | Network topology (integration) | Ranking-based method |
| Peng et al. (2015) | Network topology, sequence (integration) | Ranking-based method |
| Xiao et al. (2015) | Network topology | Ranking-based method |

* Articles found by checking the citations within the 21 retrieved articles as detailed in Section 1.

** Simple combination without any modification of the original topological feature definition.

*** The own original definition of the topological feature is modified.

## 3 Supplementary References

Acencio M. L., and Lemke N. (2009). Towards the prediction of essential genes by integration of network topology, cellular localization and biological process information. BMC Bioinformatics 10:290. doi:10.1186/1471-2105-10-290

Chen Y., and Xu D. (2005). Understanding protein dispensability through machine-learning analysis of high-throughput data. Bioinformatics. 21, 575-581. doi:10.1093/bioinformatics/bti058

Cheng, J., Wu, W., Zhang, Y., Li, X., Jiang, X., Wei, G. et al. (2013). A new computational strategy for predicting essential genes. BMC Genomics. 14:910. doi:10.1186/1471-2164-14-910

Cheng J., Xu Z., Wu W., Zhao L., Li X., Xiangchen L. et al. (2014). Training set selection for the prediction of essential genes. PLoS ONE 9:e86805. doi:10.1371/journal.pone.0086805

Deng, J., Deng, L., Su, S., Zhang, M., Lin, X., Wei, L. et al. (2011). Investigating the predictability of essential genes across distantly related organisms using an integrative approach. Nucleic Acids Res. 39, 795-807. doi:10.1093/nar/gkq784

Estrada, E. (2006). Virtual identification of essential proteins within the protein interaction network of yeast. Proteomics, 6, 35-40. doi:10.1002/pmic.200500209

Gustafson A., Snitkin E., Parker S., DeLisi C., and Kasif S. (2006). Towards the identification of essential genes using targeted genome sequencing and comparative analysis. BMC Genomics 7:265. doi:10.1186/1471-2164-7-265

Holman A., Davis P., Foster J., Carlow C., and Sabjay K. (2009). Computational prediction of essential genes in an uncluturable endosymbiotic bacterium, Wolbachia of Brugia malayi. BMC Microbiol. 9:243. doi:10.1186/1471-2180-9-243

Hwang Y. C., Lin C. C., Chang J. Y., Mori H., Juan H. F., and Huang H. C. (2009). Predicting essential genes based on network and sequence analysis. Mol. Biosyst. 5, 1672-1678. doi:10.1039/B900611G.

Jeong, H., Oltvai, Z. N., and Barabási, A. L. (2003). Prediction of protein essentiality based on genomic data. ComPlexUs, 1, 19-28. doi:10.1159/000067640

Juhas, M., Stark, M., von Mering, C., Lumjiaktase, P., Crook, D. W., Valvano, M. A. et al. (2012). High confidence prediction of essential genes in Burkholderia cenocepacia. Plos One 7:e40064. doi:0.1371/journal.pone.0040064.

Li, M., Wang, J. X., Wang, H., & Pan, Y. (2013). Identification of essential proteins from weighted protein–protein interaction networks. J Bioinform Comput Biol., 1341002. doi:10.1142/S0219720013410023

Li, M., Lu, Y., Wang, J., Wu, F. X., & Pan, Y. (2015). A topology potential-based method for identifying essential proteins from PPI networks. IEEE/ACM Trans Comput Biol Bioinform. 12, 372-383. doi:10.1109/TCBB.2014.2361350.

Liu, Y. C., Chiu, P. I., Huang, H. C., and Tseng, V. S. (2011). "Prediction of essential genes by mining gene ontology semantics" in *Bioinformatics Research and Applications* (Springer Berlin Heidelberg), 49-60. doi:10.1007/978-3-642-21260-4_9

Lu Y., Li M., Li Q., Pan Y., and Wang J. (2013). A new method for predicting essential proteins based on topology potential. In 2013 IEEE International Conference on Bioinformatics and Biomedicine, 109-114. doi:10.1109/BIBM.2013.6732472

Lu Y., Deng J., Rhodes J. C., Lu H., and Lu L. J. (2014). Predicting essential genes for identifying potential drug targets in Aspergillus fumigatus. Comput. Biol. Chem. 50, 29-40. doi:10.1016/j.compbiolchem.2014.01.011

Luo, J., and Ma, L. (2013). A New Integration-Centric Algorithm of Identifying Essential Proteins Based on Topology Structure of Protein-Protein Interaction Network and Complex Information. Curr Bioinform. 8, 380-385. doi:10.2174/1574893611308880004.

Luo J., and Kuang L. (2014). A new method for predicting essential proteins based on dynamic network topology and complex information. Comput. Biol. Chem. 52, 34-42. doi:10.1016j.compbiolchem.2014.08.022

Luo, J., and Qi, Y. (2015). Identification of Essential Proteins Based on a New Combination of Local Interaction Density and Protein Complexes. PLoS ONE, 10:e0131418. doi: 10.1371/journal.pone.0131418

Luo J., and Zhang N. (2014). Prediction of essential proteins based on edge clustering coefficient and gene ontology information. J. Biol. Syst. 22, 339-351. doi:10.1142/S0218339014500119

Peng W., Wang J. X., Wang W. P., Liu Q., Wu F. X., and Pan Y. (2012). Iteration method for predicting essential proteins based on orthology and protein-protein interaction networks. BMC Syst. Biol. 6:87. doi:10.1186/1752-0509-6-87

Peng, W., Wang, J., Cheng, Y., Lu, Y., Wu, F., & Pan, Y. (2015). UDoNC: an algorithm for identifying essential proteins based on protein domains and protein-protein interaction networks. IEEE/ACM Trans Comput Biol Bioinform. 12, 276-288. doi:10.1109/TCBB.2014.2338317

Plaimas K., Eils R., and Konig R. (2010). Identifying essential genes in bacterial metabolic networks with machine learning methods. BMC Syst. Biol. 4:56. doi:10.1186/1752-0509-4-56

Ren J., Wang J., Li M., Wang H., and Liu B. (2011). "Prediction of essential proteins by integration of PPI network topology and protein complexes information" in *Bioinformatics Research and Applications* (Springer Berlin Heidelberg), 12-24. doi:10.1007/978-3-642-21260-4_6

Saha S., and Heber S. (2006). In silico prediction of yeast deletion phenotypes. Genet. Mol. Res. 5, 224-232.

Sarangi A. N., Lohani M., and Aggarwal R. (2013). Prediction of essential proteins in prokaryotes by incorporating various physico-chemical features into the general form of Chou's pseudo amino acid composition. Protein Pept. Lett. 20, 781-795. doi:10.2174/0929866511320070008#sthash.fcoXLpym.dpuf

Seringhaus M., Paccanaro A., Borneman A., Snyder M., and Gerstein M. (2006). Predicting essential genes in fungal genomes. Genome Res. 16, 1126–1135. doi:10.1101/gr.5144106

Silva J. P. M., Acencio M. L., Mombach J. C. M., Vieira R., Silva J. C., Siniaglia M., and Lemke N. (2008). In silico network topology-based prediction of gene essentiality. Physica A 387, 1049-1055. doi:10.1016/j.physa.2007.10.044

Song, K., Tong, T., and Wu, F. (2014). Predicting essential genes in prokaryotic genomes using a linear method: ZUPLS. Integr. Biol. 6, 460-469. doi:10.1039/c3ib40241j

Tang X., Wang J., Zhong J., and Pan Y. (2014). Predicting essential proteins based on weighted degree centrality. IEEE/ACM Trans. Comput. Biol. Bioinform. 11, 407-418. doi:10.1109/TCBB.2013.2295318

Xiao, Q., Wang, J., Peng, X., Wu, F. X., and Pan, Y. (2015). Identifying essential proteins from active PPI networks constructed with dynamic gene expression. BMC Genomics, 16:S1. doi:10.1186/1471-2164-16-S3-S1

Wang, Y., Sun, H., Du, W., Blanzieri, E., Viero, G., Xu, Y., and Liang, Y. (2014). Identification of Essential Proteins Based on Ranking Edge-Weights in Protein-Protein Interaction Networks. PLoS ONE, 9:e108716. doi:10.1371/journal.pone.0108716

Zhao B., Wang J., Li M., Wu F. X., and Pan, Y. (2014). Prediction of essential proteins based on overlapping essential modules. IEEE Trans. NanoBioscience 13, 415-424. doi:10.1109/TNB.2014.2337912

Zhong J. C., Wang J. X., Peng W., Zhang Z., and Pan Y. (2013). Prediction of essential proteins based on gene expression programming. BMC Genomics 14:S7. doi:10.1186/1471-2164-14-S4-S7

## 4 List of the 38 retrieved articles

Close

**Web of Science™**
**Page 1 (Records 1 -- 38)**
[ 1 ]

Pr

**Record 1 of 38**
**By:** Jeong, Hawoong; Oltvai, Zoltan N.; Barabasi, Albert-Laszlo
**Title:** Prediction of protein essentiality based on genomic data.
**Source:** ComPlexUs
**Volume:** 1
**Issue:** 1
**Pages:** 19-28
**DOI:** 10.1159/000067640
**Published:** 2003
**Abstract:** A major goal of pharmaceutical bioinformatics is to develop computational tools for systematic in silico molecular target identification. Here we demonstrate t in the yeast Saccharomyces cerevisiae the phenotypic effect of single gene deletions simultaneously correlates with fluctuations in mRNA expression profiles, the functic categorization of the gene products, and their connectivity in the yeast's protein-protein interaction network. Building on these quantitative correlations, we developed a computational method for predicting the phenotypic effect of a given gene's functional disabling or removal. Our subsequent analyses were in good agreement with the results of systematic gene deletion experiments, allowing us to predict the deletion phenotype of a number of untested yeast genes. The results underscore the utility of la genomic databases for in silico systematic drug target identification in the postgenomic era.
**Times Cited in Web of Science Core Collection:** 43
**Times Cited in BIOSIS Citation Index:** 32
**Times Cited in Chinese Science Citation Database:** 2
**Times Cited in SciELO Citation Index:** 0
**Total Times Cited:** 48
**ISSN:** 1424-8492
**Accession Number:** BIOABS:BACD200300182536

**Record 2 of 38**
**By:** Lamichhane, G (Lamichhane, G); Zignol, M (Zignol, M); Blades, NJ (Blades, NJ); Geiman, DE (Geiman, DE); Dougherty, A (Dougherty, A); Grosset, J (Grosset, J) Broman, KW (Broman, KW); Bishai, WR (Bishai, WR)
**Author Identifiers:**

| Author | ResearcherID Number | ORCID Number |
|---|---|---|
| Broman, Karl | | 0000-0002-4914-6671 |

**Title:** A postgenomic method for predicting essential genes at subsaturation levels of mutagenesis: Application to Mycobacterium tuberculosis
**Source:** PROCEEDINGS OF THE NATIONAL ACADEMY OF SCIENCES OF THE UNITED STATES OF AMERICA
**Volume:** 100
**Issue:** 12
**Pages:** 7213-7218
**DOI:** 10.1073/pnas.1231432100
**Published:** JUN 10 2003
**Abstract:** We describe a postgenomic in silico approach for identifying genes that are likely to be essential and estimate their proportion in haploid genomes. With the knowledge of all sites eligible for mutagenesis and an experimentally determined partial list of nonessential genes from genome mutagenesis, a Bayesian statistical metho provides reasonable predictions of essential genes with a subsaturation level of random mutagenesis. For mutagenesis, a transposon such as Himar1 is suitable as it insert randomly into TA sites. All of the possible insertion sites may be determined a priori from the genome sequence and with this information, data on experimentally hit TA sites may be used to predict the proportion of genes that cannot be mutated. As a model, we used the Mycobacterium tuberculosis genome. Using the Himar1 transposon, created a genetically defined collection of 1,425 insertion mutants. Based on our Bayesian statistical analysis using Markov chain Monte Carlo and the observed frequenc of transposon insertions in all of the genes, we estimated that the M. tuberculosis genome contains 35% (95% confidence interval, 28%-41%) essential genes. This analys further revealed seven functional groups with high probabilities of being enriched in essential genes. The PE-PGRS (Pro-Glu polymorphic GC-rich repetitive sequence) family of genes, which are unique to mycobacteria, the polyketide/nonribosomal peptide synthase family, and mycolic and fatty acid biosynthesis gene families were disproportionately enriched in essential genes. At subsaturation levels of mutagenesis with a random transposon such as Himar1, this approach permits a statistical predic of both the proportion and identities of essential genes of sequenced genomes.
**Times Cited in Web of Science Core Collection:** 177
**Times Cited in BIOSIS Citation Index:** 177
**Times Cited in Chinese Science Citation Database:** 2
**Times Cited in Russian Science Citation Index:** 0
**Times Cited in SciELO Citation Index:** 1
**Total Times Cited:** 194
**ISSN:** 0027-8424
**Accession Number:** WOS:000183493500058
**PubMed ID:** 12775759

**Record 3 of 38**
**By:** Estrada, E (Estrada, E)
**Author Identifiers:**

| Author | ResearcherID Number | ORCID Number |
|---|---|---|
| Estrada, Ernesto | D-1620-2011 | 0000-0002-3066-7418 |

**Title:** Virtual identification of essential proteins within the protein interaction network of yeast
**Source:** PROTEOMICS
**Volume:** 6
**Issue:** 1
**Pages:** 35-40
**DOI:** 10.1002/pmic.200500209
**Published:** JAN 2006
**Abstract:** Topological analysis of large scale protein-protein interaction networks (PINs) is important for understanding the organizational and functional principles of individual proteins. The number of interactions that a protein has in a PIN has been observed to be correlated with its indispensability Essential proteins generally have m interactions than the nonessential ones. We show here that the lethality associated with removal of a protein from the yeast proteome correlates with different centrality measures of the nodes in the PIN, such as the closeness of a protein to many other proteins, or the number of pairs of proteins which need a specific protein as an intermediary in their communications, or the participation of a protein in different protein clusters in the PIN. These measures are significantly better than random selecti identifying essential proteins in a PIN. Centrality measures based on graph spectral properties of the network, in particular the subgraph centrality, show the best perform in identifying essential proteins in the yeast PIN. Subgraph centrality gives important structural information about the role of individual proteins, and permits the selectio possible targets for rational drug discovery through the identification of essential proteins in the PIN.
**Times Cited in Web of Science Core Collection:** 110
**Times Cited in BIOSIS Citation Index:** 71
**Times Cited in Chinese Science Citation Database:** 3
**Times Cited in Russian Science Citation Index:** 0
**Times Cited in SciELO Citation Index:** 0
**Total Times Cited:** 113
**ISSN:** 1615-9853
**Accession Number:** WOS:000234764800005
**PubMed ID:** 16281187

---

**Record 4 of 38**
**By:** Seringhaus, M (Seringhaus, Michael); Paccanaro, A (Paccanaro, Alberto); Borneman, A (Borneman, Anthony); Snyder, M (Snyder, Michael); Gerstein, M (Gerstein, Mark)
**Title:** Predicting essential genes in fungal genomes
**Source:** GENOME RESEARCH
**Volume:** 16
**Issue:** 9
**Pages:** 1126-1135
**DOI:** 10.1101/gr.5144106
**Published:** SEP 2006
**Abstract:** Essential genes are required for an organism's viability, and the ability to identify these genes in pathogens is crucial to directed drug development. Predicting essential genes through computational methods is appealing because it circumvents expensive and difficult experimental screens. Most such prediction is based on homol mapping to experimentally verified essential genes in model organisms. We present here a different approach, one that relies exclusively on sequence features of a gene t estimate essentiality and offers a promising way to identify essential genes in unstudied or uncultured organisms. We identified 14 characteristic sequence features potent associated with essentiality, such as localization signals, codon adaptation, GC content, and overall hydrophobicity. Using the well- characterized baker's yeast Saccharomyces cerevisiae, we employed a simple Bayesian framework to measure the correlation of each of these features with essentiality. We then employed the 14 features to learn the parameters of a machine learning classifier capable of predicting essential genes. We trained our classifier on known essential genes in S. cerevisiae a applied it to the closely related and relatively unstudied yeast Saccharomyces mikatae. We assessed predictive success in two ways: First, we compared all of our predicti with those generated by homology mapping between these two species. Second, we verified a subset of our predictions with eight in vivo knockouts in S. mikatae, and w present here the first experimentally confirmed essential genes in this species.
**Times Cited in Web of Science Core Collection:** 48
**Times Cited in BIOSIS Citation Index:** 43
**Times Cited in Chinese Science Citation Database:** 1
**Times Cited in Russian Science Citation Index:** 0
**Times Cited in SciELO Citation Index:** 0
**Total Times Cited:** 50
**ISSN:** 1088-9051
**Accession Number:** WOS:000240238600007
**PubMed ID:** 16899653

---

**Record 5 of 38**
**By:** Gustafson, AM (Gustafson, Adam M.); Snitkin, ES (Snitkin, Evan S.); Parker, SCJ (Parker, Stephen C. J.); DeLisi, C (DeLisi, Charles); Kasif, S (Kasif, Simon)
**Title:** Towards the identification of essential genes using targeted genome sequencing and comparative analysis
**Source:** BMC GENOMICS
**Volume:** 7
**Article Number:** 265
**DOI:** 10.1186/1471-2164-7-265
**Published:** OCT 19 2006
**Abstract:** Background: The identification of genes essential for survival is of theoretical importance in the understanding of the minimal requirements for cellular life, ar practical importance in the identification of potential drug targets in novel pathogens. With the great time and expense required for experimental studies aimed at constru a catalog of essential genes in a given organism, a computational approach which could identify essential genes with high accuracy would be of great value.
Results: We gathered numerous features which could be generated automatically from genome sequence data and assessed their relationship to essentiality, and subseque utilized machine learning to construct an integrated classifier of essential genes in both S. cerevisiae and E. coli. When looking at single features, phyletic retention, a

measure of the number of organisms an ortholog is present in, was the most predictive of essentiality. Furthermore, during construction of our phyletic retention feature w for the first time explored the evolutionary relationship among the set of organisms in which the presence of a gene is most predictive of essentiality. We found that in bot coli and S. cerevisiae the optimal sets always contain host-associated organisms with small genomes which are closely related to the reference. Using five optimally selec organisms, we were able to improve predictive accuracy as compared to using all available sequenced organisms. We hypothesize the predictive power of these genomes consequence of the process of reductive evolution, by which many parasites and symbionts evolved their gene content. In addition, essentiality is measured in rich media, condition which resembles the environments of these organisms in their hosts where many nutrients are provided. Finally, we demonstrate that integration of our most hig predictive features using a probabilistic classifier resulted in accuracies surpassing any individual feature.
Conclusion: Using features obtainable directly from sequence data, we were able to construct a classifier which can predict essential genes with high accuracy. Furthermo our analysis of the set of genomes in which the presence of a gene is most predictive of essentiality may suggest ways in which targeted sequencing can be used in the identification of essential genes. In summary, the methods presented here can aid in the reduction of time and money invested in essential gene identification by targeting those genes for experimentation which are predicted as being essential with a high probability.
**Times Cited in Web of Science Core Collection:** 53
**Times Cited in BIOSIS Citation Index:** 44
**Times Cited in Chinese Science Citation Database:** 1
**Times Cited in Russian Science Citation Index:** 0
**Times Cited in SciELO Citation Index:** 0
**Total Times Cited:** 54
**ISSN:** 1471-2164
**Accession Number:** WOS:000241743000001
**PubMed ID:** 17052348

---

**Record 6 of 38**
**By:** da Silva, JPM (Muller da Silva, Joao Paulo); Acencio, ML (Acencio, Marcio Luis); Mornbach, JCM (Merino Mornbach, Jose Carlos); Vieira, R (Vieira, Renata); da Silva, JC (da Silva, Jose Camargo); Lemke, N (Lemke, Ney); Sinigagliac, M (Sinigagliac, Marialva)
**Author Identifiers:**

| Author | ResearcherID Number | ORCID Number |
|---|---|---|
| Lemke, Ney | A-8213-2008 | 0000-0001-7463-4303 |
| Acencio, Marcio | D-9264-2012 | 0000-0002-8278-240X |

**Title:** In silico network topology-based prediction of gene essentiality
**Source:** PHYSICA A-STATISTICAL MECHANICS AND ITS APPLICATIONS
**Volume:** 387
**Issue:** 4
**Pages:** 1049-1055
**DOI:** 10.1016/j.physa.2007.10.044
**Published:** FEB 1 2008
**Abstract:** The identification of genes essential for survival is important for the understanding of the minimal requirements for cellular life and for drug design. As experimental studies with the purpose of building a catalog of essential genes for a given organism are time-consuming and laborious, a computational approach which co predict gene essentiality with high accuracy would be of great value. We present here a novel computational approach, called NTPGE (Network Topology-based Predicti of Gene Essentiality), that relies on the network topology features of a gene to estimate its essentiality. The first step of NTPGE is to construct the integrated molecular network for a given organism comprising protein physical, metabolic and transcriptional regulation interactions. The second step consists in training a decision-tree-basec machine-learning algorithm on known essential and non-essential genes of the organism of interest, considering as learning attributes the network topology information fo each of these genes. Finally, the decision-tree classifier generated is applied to the set of genes of this organism to estimate essentiality for each gene. We applied the NTI approach for discovering the essential genes in Escherichia coli and then assessed its performance. (C) 2007 Elsevier B.V. All rights reserved.
**Times Cited in Web of Science Core Collection:** 6
**Times Cited in BIOSIS Citation Index:** 6
**Times Cited in Chinese Science Citation Database:** 0
**Times Cited in Russian Science Citation Index:** 0
**Times Cited in SciELO Citation Index:** 0
**Total Times Cited:** 6
**ISSN:** 0378-4371
**Accession Number:** WOS:000252613300029

---

**Record 7 of 38**
**By:** Hwang, YC (Hwang, Yih-Chii); Lin, CC (Lin, Chen-Ching); Chang, JY (Chang, Jen-Yun); Mori, H (Mori, Hirotada); Juan, HF (Juan, Hsueh-Fen); Huang, HC (Huar Hsuan-Cheng)
**Author Identifiers:**

| Author | ResearcherID Number | ORCID Number |
|---|---|---|
| Mori, Hirotada | B-4934-2011 | |
| Huang, Hsuan-Cheng | C-7266-2011 | 0000-0002-3386-0934 |

**Title:** Predicting essential genes based on network and sequence analysis
**Source:** MOLECULAR BIOSYSTEMS
**Volume:** 5
**Issue:** 12
**Pages:** 1672-1678
**DOI:** 10.1039/b900611g
**Published:** 2009

**Abstract:** Essential genes are indispensable to the viability of an organism. Identification and analysis of essential genes is key to understanding the systems level organization of living cells. On the other hand, the ability to predict these genes in pathogens is of great importance for directed drug development. Global analysis of prc interaction networks provides an effective way to elucidate the relationships between genes. It has been found that essential genes tend to be highly connected and genera have more interactions than nonessential ones. With recent large-scale identifications of essential genes and protein-protein interactions in Saccharomyces cerevisiae and Escherichia coli, we have systematically investigated the topological properties of essential and nonessential genes in the protein-protein interaction networks. Essential genes tend to play topologically more important roles in protein interaction networks. Many topological features were found to be statistically discriminative between essential and nonessential genes. In addition, we have also examined sequence properties such as open reading frame length, strand, and phyletic retention for their association with the gene essentiality. Employing the topological features in the protein interaction network and the sequence properties, we have built a machine learning classifier capable of predicting essential genes. Computational prediction of essential genes circumvents expensive and difficult experimental screens and will help antimicrobial drug development.

**Times Cited in Web of Science Core Collection:** 29
**Times Cited in BIOSIS Citation Index:** 27
**Times Cited in Chinese Science Citation Database:** 0
**Times Cited in Russian Science Citation Index:** 0
**Times Cited in SciELO Citation Index:** 0
**Total Times Cited:** 29
**ISSN:** 1742-206X
**Accession Number:** WOS:000271727600025
**PubMed ID:** 19452048

---

**Record 8 of 38**
**By:** Martelli, C (Martelli, Carlotta); De Martino, A (De Martino, Andrea); Marinari, E (Marinari, Enzo); Marsili, M (Marsili, Matteo); Castillo, IP (Castillo, Isaac Perez)
**Author Identifiers:**

| Author | ResearcherID Number | ORCID Number |
|---|---|---|
| Marinari, Enzo | D-9124-2011 | 0000-0002-3464-4133 |

**Title:** Identifying essential genes in Escherichia coli from a metabolic optimization principle
**Source:** PROCEEDINGS OF THE NATIONAL ACADEMY OF SCIENCES OF THE UNITED STATES OF AMERICA
**Volume:** 106
**Issue:** 8
**Pages:** 2607-2611
**DOI:** 10.1073/pnas.0813229106
**Published:** FEB 24 2009
**Abstract:** Understanding the organization of reaction fluxes in cellular metabolism from the stoichiometry and the topology of the underlying biochemical network is a central issue in systems biology. In this task, it is important to devise reasonable approximation schemes that rely on the stoichiometric data only, because full-scale kinet approaches are computationally affordable only for small networks (e. g., red blood cells, approximate to 50 reactions). Methods commonly used are based on finding the stationary flux configurations that satisfy mass-balance conditions for metabolites, often coupling them to local optimization rules (e. g., maximization of biomass production) to reduce the size of the solution space to a single point. Such methods have been widely applied and have proven able to reproduce experimental findings fo relatively simple organisms in specific conditions. Here, we define and study a constraint-based model of cellular metabolism where neither mass balance nor flux stationarity are postulated and where the relevant flux configurations optimize the global growth of the system. In the case of Escherichia coli, steady flux states are recovered as solutions, although mass-balance conditions are violated for some metabolites, implying a nonzero net production of the latter. Such solutions furthermore tu out to provide the correct statistics of fluxes for the bacterium E. coli in different environments and compare well with the available experimental evidence on individual fluxes. Conserved metabolic pools play a key role in determining growth rate and flux variability. Finally, we are able to connect phenomenological gene essentiality wit "frozen" fluxes (i.e., fluxes with smaller allowed variability) in E. coli metabolism.

**Times Cited in Web of Science Core Collection:** 25
**Times Cited in BIOSIS Citation Index:** 15
**Times Cited in Chinese Science Citation Database:** 1
**Times Cited in Russian Science Citation Index:** 0
**Times Cited in SciELO Citation Index:** 0
**Total Times Cited:** 26
**ISSN:** 0027-8424
**Accession Number:** WOS:000263652900027
**PubMed ID:** 19196991

---

**Record 9 of 38**
**By:** Acencio, ML (Acencio, Marcio L.); Lemke, N (Lemke, Ney)
**Author Identifiers:**

| Author | ResearcherID Number | ORCID Number |
|---|---|---|
| Acencio, Marcio | D-9264-2012 | 0000-0002-8278-240X |
| Lemke, Ney | A-8213-2008 | 0000-0001-7463-4303 |

**Title:** Towards the prediction of essential genes by integration of network topology, cellular localization and biological process information
**Source:** BMC BIOINFORMATICS
**Volume:** 10
**Article Number:** 290
**DOI:** 10.1186/1471-2105-10-290
**Published:** SEP 16 2009

**Abstract:** Background: The identification of essential genes is important for the understanding of the minimal requirements for cellular life and for practical purposes, su as drug design. However, the experimental techniques for essential genes discovery are labor-intensive and time-consuming. Considering these experimental constraints, computational approach capable of accurately predicting essential genes would be of great value. We therefore present here a machine learning-based computational approach relying on network topological features, cellular localization and biological process information for prediction of essential genes.
Results: We constructed a decision tree-based meta-classifier and trained it on datasets with individual and grouped attributes-network topological features, cellular compartments and biological processes-to generate various predictors of essential genes. We showed that the predictors with better performances are those generated by datasets with integrated attributes. Using the predictor with all attributes, i.e., network topological features, cellular compartments and biological processes, we obtained t best predictor of essential genes that was then used to classify yeast genes with unknown essentiality status. Finally, we generated decision trees by training the J48 algori on datasets with all network topological features, cellular localization and biological process information to discover cellular rules for essentiality. We found that the num of protein physical interactions, the nuclear localization of proteins and the number of regulating transcription factors are the most important factors determining gene essentiality.
Conclusion: We were able to demonstrate that network topological features, cellular localization and biological process information are reliable predictors of essential ge Moreover, by constructing decision trees based on these data, we could discover cellular rules governing essentiality.
**Times Cited in Web of Science Core Collection:** 47
**Times Cited in BIOSIS Citation Index:** 41
**Times Cited in Chinese Science Citation Database:** 0
**Times Cited in Russian Science Citation Index:** 0
**Times Cited in SciELO Citation Index:** 0
**Total Times Cited:** 47
**ISSN:** 1471-2105
**Accession Number:** WOS:000270276400001
**PubMed ID:** 19758426

---

**Record 10 of 38**
**By:** Holman, AG (Holman, Alexander G.); Davis, PJ (Davis, Paul J.); Foster, JM (Foster, Jeremy M.); Carlow, CKS (Carlow, Clotilde K. S.); Kumar, S (Kumar, Sanjay)
**Title:** Computational prediction of essential genes in an unculturable endosymbiotic bacterium, Wolbachia of Brugia malayi
**Source:** BMC MICROBIOLOGY
**Volume:** 9
**Article Number:** 243
**DOI:** 10.1186/1471-2180-9-243
**Published:** NOV 28 2009
**Abstract:** Background: Wolbachia (wBm) is an obligate endosymbiotic bacterium of Brugia malayi, a parasitic filarial nematode of humans and one of the causative age of lymphatic filariasis. There is a pressing need for new drugs against filarial parasites, such as B. malayi. As wBm is required for B. malayi development and fertility, targeting wBm is a promising approach. However, the lifecycle of neither B. malayi nor wBm can be maintained in vitro. To facilitate selection of potential drug targets v computationally ranked the wBm genome based on confidence that a particular gene is essential for the survival of the bacterium.
Results: wBm protein sequences were aligned using BLAST to the Database of Essential Genes (DEG) version 5.2, a collection of 5,260 experimentally identified essent genes in 15 bacterial strains. A confidence score, the Multiple Hit Score (MHS), was developed to predict each wBm gene's essentiality based on the top alignments to essential genes in each bacterial strain. This method was validated using a jackknife methodology to test the ability to recover known essential genes in a control genome second estimation of essentiality, the Gene Conservation Score (GCS), was calculated on the basis of phyletic conservation of genes across Wolbachia's parent order Rickettsiales. Clusters of orthologous genes were predicted within the 27 currently available complete genomes. Druggability of wBm proteins was predicted by alignme a database of protein targets of known compounds.
Conclusion: Ranking wBm genes by either MHS or GCS predicts and prioritizes potentially essential genes. Comparison of the MHS to GCS produces quadrants representing four types of predictions: those with high confidence of essentiality by both methods (245 genes), those highly conserved across Rickettsiales (299 genes), tl similar to distant essential genes (8 genes), and those with low confidence of essentiality (253 genes). These data facilitate selection of wBm genes for entry into drug des pipelines.
**Times Cited in Web of Science Core Collection:** 46
**Times Cited in BIOSIS Citation Index:** 43
**Times Cited in Chinese Science Citation Database:** 0
**Times Cited in Russian Science Citation Index:** 0
**Times Cited in SciELO Citation Index:** 0
**Total Times Cited:** 47
**ISSN:** 1471-2180
**Accession Number:** WOS:000272823200001
**PubMed ID:** 19943957

---

**Record 11 of 38**
**By:** Plaimas, K (Plaimas, Kitiporn); Eils, R (Eils, Roland); Konig, R (Koenig, Rainer)
**Author Identifiers:**

| Author | ResearcherID Number | ORCID Number |
|---|---|---|
| Eils, Roland | B-6121-2009 | |
| Plaimas, Kitiporn | | 0000-0002-0416-8316 |

**Title:** Identifying essential genes in bacterial metabolic networks with machine learning methods
**Source:** BMC SYSTEMS BIOLOGY
**Volume:** 4
**Article Number:** 56
**DOI:** 10.1186/1752-0509-4-56
**Published:** MAY 3 2010

**Abstract:** Background: Identifying essential genes in bacteria supports to identify potential drug targets and an understanding of minimal requirements for a synthetic cel
However, experimentally assaying the essentiality of their coding genes is resource intensive and not feasible for all bacterial organisms, in particular if they are infective
Results: We developed a machine learning technique to identify essential genes using the experimental data of genome-wide knock-out screens from one bacterial organis
to infer essential genes of another related bacterial organism. We used a broad variety of topological features, sequence characteristics and co-expression properties potentially associated with essentiality, such as flux deviations, centrality, codon frequencies of the sequences, co-regulation and phyletic retention. An organism-wise cro
validation on bacterial species yielded reliable results with good accuracies (area under the receiver-operator-curve of 75%-81%). Finally, it was applied to drug target predictions for Salmonella typhimurium. We compared our predictions to the viability of experimental knock-outs of S. typhimurium and identified 35 enzymes, which a
highly relevant to be considered as potential drug targets. Specifically, we detected promising drug targets in the non-mevalonate pathway.
Conclusions: Using elaborated features characterizing network topology, sequence information and microarray data enables to predict essential genes from a bacterial reference organism to a related query organism without any knowledge about the essentiality of genes of the query organism. In general, such a method is beneficial for inferring drug targets when experimental data about genome-wide knockout screens is not available for the investigated organism.

**Times Cited in Web of Science Core Collection:** 23
**Times Cited in BIOSIS Citation Index:** 23
**Times Cited in Chinese Science Citation Database:** 1
**Times Cited in Russian Science Citation Index:** 0
**Times Cited in SciELO Citation Index:** 0
**Total Times Cited:** 24
**ISSN:** 1752-0509
**Accession Number:** WOS:000278258200001
**PubMed ID:** 20438628

---

**Record 12 of 38**
**By:** Ren, J (Ren, Jun); Wang, JX (Wang, Jianxin); Li, M (Li, Min); Wang, H (Wang, Huan); Liu, BB (Liu, Binbin)
**Edited by:** Chen, J (Chen, J); Wang, JX (Wang, JX); Zelikovsky, A (Zelikovsky, A)
**Title:** Prediction of Essential Proteins by Integration of PPI Network Topology and Protein Complexes Information
**Source:** BIOINFORMATICS RESEARCH AND APPLICATIONS
**Book Series Title:** Lecture Notes in Bioinformatics
**Volume:** 6674
**Pages:** 12-24
**Published:** 2011
**Abstract:** Identifying essential proteins is important for understanding the minimal requirements for cellular survival and development. Numerous computational methoc
have been proposed to identify essential proteins from protein-protein interaction (PPI) network. However most of methods only use the PPI network topology informatic
HartGT indicated that essentiality is a product of the protein complex rather than the individual protein. Based on these, we propose a new method ECC to identify essent
proteins by integration of subgraph centrality (SC) of PPI network and protein complexes information. We apply ECC method and six centrality methods on the yeast PP
network. The experimental results show that the performance of ECC is much better than that of six centrality methods, which means that the prediction of essential prote
based on both network topology and protein complexes set is much better than that only based on network topology. Moreover. ECC has a significant improvement in prediction of low-connectivity essential proteins.
**Conference Title:** 7th International Symposium on Bioinformatics Research and Applications (ISBRA)
**Conference Date:** MAY 27-29, 2011
**Conference Location:** Cent S Univ, Changsha, PEOPLES R CHINA
**Conference Host:** Cent S Univ
**Times Cited in Web of Science Core Collection:** 13
**Times Cited in BIOSIS Citation Index:** 12
**Times Cited in Chinese Science Citation Database:** 0
**Times Cited in Russian Science Citation Index:** 0
**Times Cited in SciELO Citation Index:** 0
**Total Times Cited:** 13
**ISSN:** 0302-9743
**ISBN:** 978-3-642-21259-8
**Accession Number:** WOS:000296688700006

---

**Record 13 of 38**
**By:** Juhas, M (Juhas, Mario); Stark, M (Stark, Manuel); von Mering, C (von Mering, Christian); Lumjiaktase, P (Lumjiaktase, Puthapoom); Crook, DW (Crook, Derrick
Valvano, MA (Valvano, Miguel A.); Eberl, L (Eberl, Leo)
**Author Identifiers:**

| Author | ResearcherID Number | ORCID Number |
|---|---|---|
| von Mering, Christian | B-3300-2008 | 0000-0001-7734-9102 |
| Valvano, Miguel | G-8759-2012 | |
| Valvano, Miguel | | 0000-0001-8229-3641 |

**Title:** High Confidence Prediction of Essential Genes in Burkholderia Cenocepacia
**Source:** PLOS ONE
**Volume:** 7
**Issue:** 6
**Article Number:** e40064
**DOI:** 10.1371/journal.pone.0040064
**Published:** JUN 29 2012

**Abstract:** Background: Essential genes are absolutely required for the survival of an organism. The identification of essential genes, besides being one of the most fundamental questions in biology, is also of interest for the emerging science of synthetic biology and for the development of novel antimicrobials. New antimicrobial therapies are desperately needed to treat multidrug-resistant pathogens, such as members of the Burkholderia cepacia complex.
Methodology/Principal Findings: We hypothesize that essential genes may be highly conserved within a group of evolutionary closely related organisms. Using a bioinformatics approach we determined that the core genome of the order Burkholderiales consists of 649 genes. All but two of these identified genes were located on chromosome 1 of Burkholderia cenocepacia. Although many of the 649 core genes of Burkholderiales have been shown to be essential in other bacteria, we were also abl identify a number of novel essential genes present mainly, or exclusively, within this order. The essentiality of some of the core genes, including the known essential gene infB, gyrB, ubiB, and valS, as well as the so far uncharacterized genes BCAL1882, BCAL2769, BCAL3142 and BCAL3369 has been confirmed experimentally in B. cenocepacia.
Conclusions/Significance: We report on the identification of essential genes using a novel bioinformatics strategy and provide bioinformatics and experimental evidence t the large majority of the identified genes are indeed essential. The essential genes identified here may represent valuable targets for the development of novel antimicrobi and their detailed study may shed new light on the functions required to support life.
**Times Cited in Web of Science Core Collection:** 20
**Times Cited in BIOSIS Citation Index:** 18
**Times Cited in Chinese Science Citation Database:** 0
**Times Cited in Russian Science Citation Index:** 0
**Times Cited in SciELO Citation Index:** 0
**Total Times Cited:** 20
**ISSN:** 1932-6203
**Accession Number:** WOS:000305892100193
**PubMed ID:** 22768221

---

**Record 14 of 38**
**By:** Peng, W (Peng, Wei); Wang, JX (Wang, Jianxin); Wang, WP (Wang, Weiping); Liu, Q (Liu, Qing); Wu, FX (Wu, Fang-Xiang); Pan, Y (Pan, Yi)
**Title:** Iteration method for predicting essential proteins based on orthology and protein-protein interaction networks
**Source:** BMC SYSTEMS BIOLOGY
**Volume:** 6
**Article Number:** 87
**DOI:** 10.1186/1752-0509-6-87
**Published:** JUL 18 2012
**Abstract:** Background: Identification of essential proteins plays a significant role in understanding minimal requirements for the cellular survival and development. Man computational methods have been proposed for predicting essential proteins by using the topological features of protein-protein interaction (PPI) networks. However, mo these methods ignored intrinsic biological meaning of proteins. Moreover, PPI data contains many false positives and false negatives. To overcome these limitations, rece many research groups have started to focus on identification of essential proteins by integrating PPI networks with other biological information. However, none of their methods has widely been acknowledged.
Results: By considering the facts that essential proteins are more evolutionarily conserved than nonessential proteins and essential proteins frequently bind each other, we propose an iteration method for predicting essential proteins by integrating the orthology with PPI networks, named by ION. Differently from other methods, ION identif essential proteins depending on not only the connections between proteins but also their orthologous properties and features of their neighbors. ION is implemented to pr essential proteins in S. cerevisiae. Experimental results show that ION can achieve higher identification accuracy than eight other existing centrality methods in terms of under the curve (AUC). Moreover, ION identifies a large amount of essential proteins which have been ignored by eight other existing centrality methods because of thei low-connectivity. Many proteins ranked in top 100 by ION are both essential and belong to the complexes with certain biological functions. Furthermore, no matter how many reference organisms were selected, ION outperforms all eight other existing centrality methods. While using as many as possible reference organisms can improve performance of ION. Additionally, ION also shows good prediction performance in E. coli K-12.
Conclusions: The accuracy of predicting essential proteins can be improved by integrating the orthology with PPI networks.
**Times Cited in Web of Science Core Collection:** 22
**Times Cited in BIOSIS Citation Index:** 19
**Times Cited in Chinese Science Citation Database:** 1
**Times Cited in Russian Science Citation Index:** 0
**Times Cited in SciELO Citation Index:** 0
**Total Times Cited:** 23
**ISSN:** 1752-0509
**Accession Number:** WOS:000310442200001
**PubMed ID:** 22808943

---

**Record 15 of 38**
**By:** Deng, JY (Deng, Jingyuan); Deng, L (Deng, Lei); Su, SC (Su, Shengchang); Zhang, ML (Zhang, Minlu); Lin, XD (Lin, Xiaodong); Wei, L (Wei, Lan); Minai, AA (Minai, Ali A.); Hassett, DJ (Hassett, Daniel J.); Lu, LJ (Lu, Long J.)
**Title:** Investigating the predictability of essential genes across distantly related organisms using an integrative approach (vol 39, pg 795, 2012)
**Source:** NUCLEIC ACIDS RESEARCH
**Volume:** 40
**Issue:** 16
**Pages:** 8198-8198
**DOI:** 10.1093/nar/gks814
**Published:** SEP 2012
**Times Cited in Web of Science Core Collection:** 0
**Times Cited in BIOSIS Citation Index:** 0
**Times Cited in Chinese Science Citation Database:** 0
**Times Cited in Russian Science Citation Index:** 0
**Times Cited in SciELO Citation Index:** 0

**Total Times Cited:** 0
**ISSN:** 0305-1048
**Accession Number:** WOS:000308959800060

---

**Record 16 of 38**
**By:** Klein, BA (Klein, Brian A.); Tenorio, EL (Tenorio, Elizabeth L.); Lazinski, DW (Lazinski, David W.); Camilli, A (Camilli, Andrew); Duncan, MJ (Duncan, Margare
Hu, LDT (Hu, Linden T.)
**Title:** Identification of essential genes of the periodontal pathogen Porphyromonas gingivalis
**Source:** BMC GENOMICS
**Volume:** 13
**Article Number:** 578
**DOI:** 10.1186/1471-2164-13-578
**Published:** OCT 31 2012
**Abstract:** Background: Porphyromonas gingivalis is a Gram-negative anaerobic bacterium associated with periodontal disease onset and progression. Genetic tools for th manipulation of bacterial genomes allow for in-depth mechanistic studies of metabolism, physiology, interspecies and host-pathogen interactions. Analysis of the essentia genes, protein-coding sequences necessary for survival of P. gingivalis by transposon mutagenesis has not previously been attempted due to the limitations of available transposon systems for the organism. We adapted a Mariner transposon system for mutagenesis of P. gingivalis and created an insertion mutant library. By analyzing the location of insertions using massively-parallel sequencing technology we used this mutant library to define genes essential for P. gingivalis survival under in vitro conditi
Results: In mutagenesis experiments we identified 463 genes in P. gingivalis strain ATCC 33277 that are putatively essential for viability in vitro. Comparing the 463 P. gingivalis essential genes with previous essential gene studies, 364 of the 463 are homologues to essential genes in other species; 339 are shared with more than one othe species. Twenty-five genes are known to be essential in P. gingivalis and B. thetaiotaomicron only. Significant enrichment of essential genes within Cluster of Orthologou Groups 'D' (cell division), 'I' (lipid transport and metabolism) and 'J' (translation/ribosome) were identified. Previously, the P. gingivalis core genome was shown to encod 1,476 proteins out of a possible 1,909; 434 of 463 essential genes are contained within the core genome. Thus, for the species P. gingivalis twenty-two, seventy-seven anc twenty-three percent of the genome respectively are devoted to essential, core and accessory functions.
Conclusions: A Mariner transposon system can be adapted to create mutant libraries in P. gingivalis amenable to analysis by next-generation sequencing technologies. In silico analysis of genes essential for in vitro growth demonstrates that although the majority are homologous across bacterial species as a whole, species and strain-specif subsets are apparent. Understanding the putative essential genes of P. gingivalis will provide insights into metabolic pathways and niche adaptations as well as clinical therapeutic strategies.
**Times Cited in Web of Science Core Collection:** 27
**Times Cited in BIOSIS Citation Index:** 26
**Times Cited in Chinese Science Citation Database:** 2
**Times Cited in Russian Science Citation Index:** 0
**Times Cited in SciELO Citation Index:** 0
**Total Times Cited:** 1981
**ISSN:** 1471-2164
**Accession Number:** WOS:000314646600001
**PubMed ID:** 23114059

---

**Record 17 of 38**
**By:** Lu, Y (Lu, Yu); Li, M (Li, Min); Li, Q (Li, Qi); Pan, Y (Pan, Yi); Wang, JX (Wang, Jianxin)
**Edited by:** Li, GZ (Li, GZ); Kim, S (Kim, S); Hughes, M (Hughes, M); McLachlan, G (McLachlan, G); Sun, H (Sun, H); Hu X (Hu, X); Ressom, H (Ressom, H); Liu, E (Liu, B); Liebman, M (Liebman, M)
**Title:** A new method for predicting essential proteins based on topology potential
**Source:** 2013 IEEE INTERNATIONAL CONFERENCE ON BIOINFORMATICS AND BIOMEDICINE (BIBM)
**Book Series Title:** IEEE International Conference on Bioinformatics and Biomedicine-BIBM
**Published:** 2013
**Abstract:** Essential proteins are indispensable for cellular life. It is of great significance to identify essential proteins that can help us understand the minimal requiremen for cellular life and is also very important for drug design. However, identification of essential proteins based on experimental approaches are always time-consuming anc expensive. With the development of high-throughput technology in the post-genomic era, more and more protein-protein interaction data can be obtained, which make us study essential proteins from the network level become possible. There have been a series of computational approaches proposed for predicting essential proteins based o network topologies. Most of these topology based essential protein discovery methods were to use network centrality. In this paper, we investigate the essential proteins' topological characters from a completely new perspective. To our knowledge it is the first time that topology potential is used to identify essential proteins from protein-protein interaction network. The basic idea is that each protein in the network can be viewed as a material particle which creates a potential field around itself and the interaction of all proteins forms a topological field over the network. By defining and computing the value of each protein's topology potential, we can obtain a more prec ranking which reflects the importance of proteins from the protein-protein interaction network. The experiment results show that topology potential outperforms tradition topology measures: Degree Centrality (DC), Betweenness Centrality (BC), Closeness Centrality (CC), Subgraph Centrality(SC), Eigenvector Centrality(EC), Informatior Centrality(IC), and Sum of ECC (NC) for predicting essential proteins. In addition, these centrality measures are improved on their performance for identifying essential proteins in biological network when controlled by topology potential.
**Conference Title:** IEEE International Conference on Bioinformatics and Biomedicine (IEEE BIBM)
**Conference Date:** DEC 18-21, 2013
**Conference Location:** Shanghai, PEOPLES R CHINA
**Sponsor(s):** IEEE; IEEE Comp Soc; Tongji Univ; IEEE Comp Soc Tech Comm Life Sci; China Acad Chinese Med Sci; Shanghai Assoc Syst Simulat
**Times Cited in Web of Science Core Collection:** 0
**Times Cited in BIOSIS Citation Index:** 0
**Times Cited in Chinese Science Citation Database:** 0
**Times Cited in Russian Science Citation Index:** 0
**Times Cited in SciELO Citation Index:** 0
**Total Times Cited:** 0
**ISSN:** 2156-1125

**ISBN:** 978-1-4799-1309-1; 978-1-4799-1310-7
**Accession Number:** WOS:000348252400025

---

**Record 18 of 38**

**By:** Wang, JX (Wang, Jianxin); Peng, W (Peng, Wei); Chen, YJ (Chen, Yingjiao); Lu, Y (Lu, Yu); Pan, Y (Pan, Yi)

**Edited by:** Li, GZ (Li, GZ); Kim, S (Kim, S); Hughes, M (Hughes, M); McLachlan, G (McLachlan, G); Sun, H (Sun, H); Hu X (Hu, X); Ressom, H (Ressom, H); Liu, B (Liu, B); Liebman, M (Liebman, M)

**Title:** Identifying essential proteins based on protein domains in protein-protein interaction networks

**Source:** 2013 IEEE INTERNATIONAL CONFERENCE ON BIOINFORMATICS AND BIOMEDICINE (BIBM)

**Book Series Title:** IEEE International Conference on Bioinformatics and Biomedicine-BIBM

**Published:** 2013

**Abstract:** Prediction of essential proteins which are crucial to an organism survival is important for disease analysis and drug design, as well as the understanding of cell life. The majority of prediction methods infer the possibility of proteins to be essential by using the network topology. However, these methods are limited to the complementation of available protein-protein interaction (PPI) data and depend on the network accuracy. To overcome these limitation, some computational methods hav been proposed while seldom of them solve this problem by taking consideration of protein domains. In this work, we firstly analyze the correlation between the essentiali proteins and their domain features based on data of 13 species. We find that the proteins containing more protein domain types which rarely occur in other proteins tend t essential. Accordingly we propose a new prediction method, named UDoNC, by combining the domain features of proteins with their topological properties in PPI netwo In UDoNC, the essentiality of proteins is decided by the number and the frequency of their protein domain types, as well as the essentiality of their adjacent edges measu by edge clustering coefficient. The experimental results on S. cerevisiae data show that UDoNC outperforms other existing methods in terms of area under the curve (AU

**Conference Title:** IEEE International Conference on Bioinformatics and Biomedicine (IEEE BIBM)

**Conference Date:** DEC 18-21, 2013

**Conference Location:** Shanghai, PEOPLES R CHINA

**Sponsor(s):** IEEE; IEEE Comp Soc; Tongji Univ; IEEE Comp Soc Tech Comm Life Sci; China Acad Chinese Med Sci; Shanghai Assoc Syst Simulat

**Times Cited in Web of Science Core Collection:** 0

**Times Cited in BIOSIS Citation Index:** 0

**Times Cited in Chinese Science Citation Database:** 0

**Times Cited in Russian Science Citation Index:** 0

**Times Cited in SciELO Citation Index:** 0

**Total Times Cited:** 0

**ISSN:** 2156-1125
**ISBN:** 978-1-4799-1309-1; 978-1-4799-1310-7
**Accession Number:** WOS:000348252400029

---

**Record 19 of 38**

**By:** Wang, JX (Wang, Jianxin); Peng, W (Peng, Wei); Wu, FX (Wu, Fang-Xiang)

**Title:** Computational approaches to predicting essential proteins: A survey

**Source:** PROTEOMICS CLINICAL APPLICATIONS

**Volume:** 7

**Issue:** 1-2

**Special Issue:** SI

**Pages:** 181-192

**DOI:** 10.1002/prca.201200068

**Published:** JAN 2013

**Abstract:** Essential proteins are indispensable to support cellular life. Identifying essential proteins can help us understand the minimal requirements for cell survival, wh plays a significant role in the emerging field of synthetic biology. Moreover, essential proteins also serve as candidates of drug targets for developing novel therapy of diseases, such as cancer or infectious disease caused by emerging pathogens. However, it is expensive and time consuming to experimentally identify essential proteins. \ accumulation of sequenced genomes, the gap between genome-wide essential protein data and sequence data become increasingly wide. Thus, computational approaches detecting essential proteins are useful complements to limited experimental methods. There are many features related to protein essentiality. By taking advantage of these features, many computational approaches have been proposed to identify essential proteins. In this paper, we review the state-of-the-art techniques for computational detection of essential proteins, and discuss some challenges for future research in this field.

**Times Cited in Web of Science Core Collection:** 20

**Times Cited in BIOSIS Citation Index:** 14

**Times Cited in Chinese Science Citation Database:** 0

**Times Cited in Russian Science Citation Index:** 0

**Times Cited in SciELO Citation Index:** 0

**Total Times Cited:** 20

**ISSN:** 1862-8346
**Accession Number:** WOS:000313903700017
**PubMed ID:** 23165920

---

**Record 20 of 38**

**By:** Hor, CY (Hor, Chiou-Yi); Yang, CB (Yang, Chang-Biau); Yang, ZJ (Yang, Zih-Jie); Tseng, CT (Tseng, Chiou-Ting)

**Title:** Prediction of Protein Essentiality by the Support Vector Machine with Statistical Tests

**Source:** EVOLUTIONARY BIOINFORMATICS

**Volume:** 9

**Pages:** 387-416

**DOI:** 10.4137/EBO.S11975

**Published:** 2013

**Abstract:** Essential proteins include the minimum required set of proteins to support cell life. Identifying essential proteins is important for understanding the cellular processes of an organism. However, identifying essential proteins experimentally is extremely time-consuming and labor-intensive. Alternative methods must be develop to examine essential proteins. There were two goals in this study: identifying the important features and building learning machines for discriminating essential proteins. for Saccharomyces cerevisiae and Escherichia coli were used. We first collected information from a variety of sources. We next proposed a modified backward feature selection method and build support vector machines (SVM) predictors based on the selected features. To evaluate the performance, we conducted cross-validations for th originally imbalanced data set and the down-sampling balanced data set. The statistical tests were applied on the performance associated with obtained feature subsets to confirm their significance. In the first data set, our best values of F-measure and Matthews correlation coefficient (MCC) were 0.549 and 0.495 in the imbalanced experiments. For the balanced experiment, the best values of F-measure and MCC were 0.770 and 0.545, respectively. In the second data set, our best values of F-measur and MCC were 0.421 and 0.407 in the imbalanced experiments. For the balanced experiment, the best values of F-measure and MCC were 0.718 and 0.448, respectively. experimental results show that our selected features are compact and the performance improved. Prediction can also be conducted by users at the following internet addre http://bio2.cse.nsysu.edu.tw/esspredict.aspx.

**Times Cited in Web of Science Core Collection:** 1
**Times Cited in BIOSIS Citation Index:** 1
**Times Cited in Chinese Science Citation Database:** 0
**Times Cited in Russian Science Citation Index:** 0
**Times Cited in SciELO Citation Index:** 0
**Total Times Cited:** 1
**ISSN:** 1176-9343
**Accession Number:** WOS:000325126800001
**PubMed ID:** 24250217

---

**Record 21 of 38**
**By:** Lakshmanan, M (Lakshmanan, Meiyappan); Mohanty, B (Mohanty, Bijayalaxmi); Lee, DY (Lee, Dong-Yup)
**Author Identifiers:**

| Author | ResearcherID Number | ORCID Number |
|---|---|---|
| Lee, Dong-Yup | D-6650-2011 | 0000-0003-0901-708X |
| Lakshmanan, Meiyappan | H-1267-2014 | 0000-0003-2356-3458 |

**Title:** Identifying essential genes/reactions of the rice photorespiration by in silico model-based analysis
**Source:** RICE
**Volume:** 6
**Article Number:** 20
**DOI:** 10.1186/1939-8433-6-20
**Published:** 2013
**Abstract:** Background: Photorespiration, a highly wasteful process of energy dissipation, depresses the productivity of C3 plants such as rice (Oryza sativa) under dry an hot conditions. Thus, it is highly required to understand the cellular physiology and relevant metabolic states under photorespiration using systems approaches, thereby devising strategies for improving rice production.
Findings: In silico model-driven gene deletion analysis was performed on photorespiring leaf cells under ambient and stressed environmental conditions using our central metabolic network of rice cells. As a result, we identified a number of essential genes for the cell growth across various functional pathways such as photorespiratory cyc Calvin cycle, GS-GOGAT cycle and sucrose metabolism as well as certain inter-compartmental transporters, which are mostly in good agreement with previous experime Synthetic lethal (SL) screening was also performed to identify the pair of non-essential genes whose simultaneous deletion become lethal, revealing the existence of more than 220 pairs of SLs on rice central metabolism.
Conclusions: The gene deletion and synthetic lethal analyses highlighted the rigid nature of rice photosynthetic pathways and characterized functional interactions betwe central metabolic genes, respectively. The biological roles of such reported essential genes should be further explored to better understand the rice photorespiration in futu
**Times Cited in Web of Science Core Collection:** 0
**Times Cited in BIOSIS Citation Index:** 1
**Times Cited in Chinese Science Citation Database:** 0
**Times Cited in Russian Science Citation Index:** 0
**Times Cited in SciELO Citation Index:** 0
**Total Times Cited:** 1
**ISSN:** 1939-8425
**eISSN:** 1939-8433
**Accession Number:** WOS:000329807100002
**PubMed ID:** 24280628

---

**Record 22 of 38**
**By:** Li, M (Li, Min); Wang, JX (Wang, Jian-Xin); Wang, H (Wang, Huan); Pan, Y (Pan, Yi)
**Title:** IDENTIFICATION OF ESSENTIAL PROTEINS FROM WEIGHTED PROTEIN-PROTEIN INTERACTION NETWORKS
**Source:** JOURNAL OF BIOINFORMATICS AND COMPUTATIONAL BIOLOGY
**Volume:** 11
**Issue:** 3
**Special Issue:** SI
**Article Number:** 1341002
**DOI:** 10.1142/S0219720013410023
**Published:** JUN 2013
**Abstract:** Identifying essential proteins is very important for understanding the minimal requirements of cellular survival and development. Fast growth in the amount of available protein-protein interactions has produced unprecedented opportunities for detecting protein essentiality on network level. A series of centrality measures have b proposed to discover essential proteins based on network topology. Unfortunately, the protein-protein interactions produced by high-throughput experiments generally ha

high false positives. Moreover, most of centrality measures based on network topology are sensitive to false positives. We therefore propose a new method for evaluating confidence of each interaction based on the combination of logistic regression-based model and function similarity. Nine standard centrality measures in weighted networ were redefined in this paper. The experimental results on a yeast protein interaction network shows that the weighting method improved the performance of centrality measures considerably. More essential proteins were discovered by the weighted centrality measures than by the original centrality measures used in the unweighted netw Even about 20% improvements were obtained from closeness centrality and subgraph centrality.

**Times Cited in Web of Science Core Collection:** 8
**Times Cited in BIOSIS Citation Index:** 6
**Times Cited in Chinese Science Citation Database:** 0
**Times Cited in Russian Science Citation Index:** 0
**Times Cited in SciELO Citation Index:** 0
**Total Times Cited:** 8
**ISSN:** 0219-7200
**Accession Number:** WOS:000321005400002
**PubMed ID:** 23796179

---

**Record 23 of 38**
**By:** Sarangi, AN (Sarangi, Aditya Narayan); Lohani, M (Lohani, Mohtashim); Aggarwal, R (Aggarwal, Rakesh)
**Author Identifiers:**

| Author | ResearcherID Number | ORCID Number |
|---|---|---|
| Aggarwal, Rakesh | D-1583-2009 | 0000-0001-9689-494X |

**Title:** Prediction of Essential Proteins in Prokaryotes by Incorporating Various Physico-chemical Features into the General form of Chou's Pseudo Amino Acid Composit
**Source:** PROTEIN AND PEPTIDE LETTERS
**Volume:** 20
**Issue:** 7
**Pages:** 781-795
**Published:** JUL 2013
**Abstract:** Prediction of essential proteins of a pathogenic organism is the key for the potential drug target identification, because inhibition of these would be fatal for the pathogen. Identification of these proteins requires the use of complex experimental techniques which are quite expensive and time consuming. We implemented Support Vector Machine algorithm to develop a classifier model for in silico prediction of prokaryotic essential proteins based on the physico-chemical properties of the amino ac sequences. This classifier was designed based on a set of 10 physico-chemical descriptor vectors (DVs) and 4 hybrid DVs calculated from amino acid sequences using PROFEAT and PseAAC servers. The classifier was trained using data sets consisting of 500 known essential and 500 non-essential proteins (n=1,000) and evaluated usin external validation set consisting of 3,462 essential proteins and 5,538 non-essential proteins (n=9,000). The performances of individual DV sets were evaluated. DV set which is the combination of composition, transition and distribution descriptor set and hybrid autocorrelation descriptor set, provided accuracy of 91.2% in 10-fold cross-validation of the training set and an accuracy of 89.7% in external validation set and of 91.8% and 88.1% using a different yeast protein dataset. Our result indicates that t classification model can be used for identification of novel prokaryotic essential proteins.
**Times Cited in Web of Science Core Collection:** 8
**Times Cited in BIOSIS Citation Index:** 5
**Times Cited in Chinese Science Citation Database:** 0
**Times Cited in Russian Science Citation Index:** 0
**Times Cited in SciELO Citation Index:** 0
**Total Times Cited:** 8
**ISSN:** 0929-8665
**eISSN:** 1875-5305
**Accession Number:** WOS:000319499400008
**PubMed ID:** 23276224

---

**Record 24 of 38**
**By:** Luo, JW (Luo, Jiawei); Ma, L (Ma, Ling)
**Title:** A New Integration-Centric Algorithm of Identifying Essential Proteins Based on Topology Structure of Protein-Protein Interaction Network and Complex Informa
**Source:** CURRENT BIOINFORMATICS
**Volume:** 8
**Issue:** 3
**Pages:** 380-385
**Published:** JUL 2013
**Abstract:** Essential proteins are necessary for the survival and development of organism. Many computational approaches have been proposed for predicting essential proteins based on protein-protein interaction (PPI) network. In this paper, we propose a new centrality algorithm for identifying essential proteins, named CSC algorithm. CSC algorithm integrates topology character of PPI network and in-degree of proteins in complexes. We use CSC algorithm to identify the essential proteins in PPI netwo of Saccharomyces cerevisiae. The results show that the ratio of identified essential proteins on CSC algorithm is higher than other ten centrality methods: Degree Central (DC), Betweenness Centrality (BC), Closeness Centrality (CC), Subgraph Centrality (SC), Eigenvector Centrality (EC), Information Centrality (IC), Bottle Neck (BN), L Average Connectivity-based method (LAC), Sum of ECC (SoECC) and PeC. Particularly, the identification accuracy of CSC algorithm is more than 40% over the six cla centrality measures (DC, BC, CC, SC, EC, IC).
**Times Cited in Web of Science Core Collection:** 1
**Times Cited in BIOSIS Citation Index:** 1
**Times Cited in Chinese Science Citation Database:** 0
**Times Cited in Russian Science Citation Index:** 0
**Times Cited in SciELO Citation Index:** 0
**Total Times Cited:** 1

**ISSN:** 1574-8936
**Accession Number:** WOS:000320467300014

---

**Record 25 of 38**

**By:** Zhong, JC (Zhong, Jiancheng); Wang, JX (Wang, Jianxin); Peng, W (Peng, Wei); Zhang, Z (Zhang, Zhen); Pan, Y (Pan, Yi)

**Title:** Prediction of essential proteins based on gene expression programming

**Source:** BMC GENOMICS
**Volume:** 14
**Article Number:** S7
**DOI:** 10.1186/1471-2164-14-S4-S7
**Supplement:** 4
**Published:** OCT 1 2013

**Abstract:** Background: Essential proteins are indispensable for cell survive. Identifying essential proteins is very important for improving our understanding the way of a working. There are various types of features related to the essentiality of proteins. Many methods have been proposed to combine some of them to predict essential protei However, it is still a big challenge for designing an effective method to predict them by integrating different features, and explaining how these selected features decide th essentiality of protein. Gene expression programming (GEP) is a learning algorithm and what it learns specifically is about relationships between variables in sets of data then builds models to explain these relationships.
Results: In this work, we propose a GEP-based method to predict essential protein by combing some biological features and topological features. We carry out experimen on S. cerevisiae data. The experimental results show that the our method achieves better prediction performance than those methods using individual features. Moreover, method outperforms some machine learning methods and performs as well as a method which is obtained by combining the outputs of eight machine learning methods.
Conclusions: The accuracy of predicting essential proteins can been improved by using GEP method to combine some topological features and biological features.

**Times Cited in Web of Science Core Collection:** 2
**Times Cited in BIOSIS Citation Index:** 1
**Times Cited in Chinese Science Citation Database:** 0
**Times Cited in Russian Science Citation Index:** 0
**Times Cited in SciELO Citation Index:** 0
**Total Times Cited:** 2

**ISSN:** 1471-2164
**Accession Number:** WOS:000326801200007
**PubMed ID:** 24267033

---

**Record 26 of 38**

**By:** Cheng, J (Cheng, Jian); Wu, WW (Wu, Wenwu); Zhang, YW (Zhang, Yinwen); Li, XC (Li, Xiangchen); Jiang, XQ (Jiang, Xiaoqian); Wei, GH (Wei, Gehong); Tao, (Tao, Shiheng)

**Title:** A new computational strategy for predicting essential genes

**Source:** BMC GENOMICS
**Volume:** 14
**Article Number:** 910
**DOI:** 10.1186/1471-2164-14-910
**Published:** DEC 21 2013

**Abstract:** Background: Determination of the minimum gene set for cellular life is one of the central goals in biology. Genome-wide essential gene identification has progressed rapidly in certain bacterial species; however, it remains difficult to achieve in most eukaryotic species. Several computational models have recently been developed to integrate gene features and used as alternatives to transfer gene essentiality annotations between organisms.
Results: We first collected features that were widely used by previous predictive models and assessed the relationships between gene features and gene essentiality using stepwise regression model. We found two issues that could significantly reduce model accuracy: (i) the effect of multicollinearity among gene features and (ii) the diverse even contrasting correlations between gene features and gene essentiality existing within and among different species. To address these issues, we developed a novel mod called feature-based weighted Naive Bayes model (FWM), which is based on Naive Bayes classifiers, logistic regression, and genetic algorithm. The proposed model assesses features and filters out the effects of multicollinearity and diversity. The performance of FWM was compared with other popular models, such as support vector machine, Naive Bayes model, and logistic regression model, by applying FWM to reciprocally predict essential genes among and within 21 species. Our results showed t FWM significantly improves the accuracy and robustness of essential gene prediction.
Conclusions: FWM can remarkably improve the accuracy of essential gene prediction and may be used as an alternative method for other classification work. This metho can contribute substantially to the knowledge of the minimum gene sets required for living organisms and the discovery of new drug targets.

**Times Cited in Web of Science Core Collection:** 1
**Times Cited in BIOSIS Citation Index:** 1
**Times Cited in Chinese Science Citation Database:** 0
**Times Cited in Russian Science Citation Index:** 0
**Times Cited in SciELO Citation Index:** 0
**Total Times Cited:** 1

**ISSN:** 1471-2164
**Accession Number:** WOS:000329367000004
**PubMed ID:** 24359534

---

**Record 27 of 38**

**By:** Li, W (Li, Wei); Xu, H (Xu, Han); Xiao, TF (Xiao, Tengfei); Cong, L (Cong, Le); Love, MI (Love, Michael I.); Zhang, F (Zhang, Feng); Irizarry, RA (Irizarry, Rafae A.); Liu, JS (Liu, Jun S.); Brown, M (Brown, Myles); Liu, XS (Liu, X. Shirley)

**Author Identifiers:**

| Author | ResearcherID Number | ORCID Number |
|---|---|---|
| Cong, Le | M-9981-2013 | 0000-0003-4725-8714 |

**Title:** MAGeCK enables robust identification of essential genes from genome-scale CRISPR/Cas9 knockout screens
**Source:** GENOME BIOLOGY
**Volume:** 15
**Issue:** 12
**Article Number:** 554
**DOI:** 10.1186/s13059-014-0554-4
**Published:** 2014
**Abstract:** We propose the Model-based Analysis of Genome-wide CRISPR/Cas9 Knockout (MAGeCK) method for prioritizing single-guide RNAs, genes and pathways genome-scale CRISPR/Cas9 knockout screens. MAGeCK demonstrates better performance compared with existing methods, identifies both positively- and negatively-selected genes simultaneously, and reports robust results across different experimental conditions. Using public datasets, MAGeCK identified novel essential genes and pathways, including EGFR in vemurafenib treated A375 cells harboring a BRAF mutation. MAGeCK also detected cell-type specific essential genes including BCR and ABL1 in the KBM7 cells bearing a BCR-ABL fusion, and IGF1R in the HL-60 cells, which depends on the insulin signaling pathway for proliferation.
**Times Cited in Web of Science Core Collection:** 3
**Times Cited in BIOSIS Citation Index:** 2
**Times Cited in Chinese Science Citation Database:** 0
**Times Cited in Russian Science Citation Index:** 0
**Times Cited in SciELO Citation Index:** 0
**Total Times Cited:** 3
**ISSN:** 1465-6906
**eISSN:** 1474-760X
**Accession Number:** WOS:000346609500026
**PubMed ID:** 25476604

---

**Record 28 of 38**
**By:** Cheng, J (Cheng, Jian); Xu, Z (Xu, Zhao); Wu, WW (Wu, Wenwu); Zhao, L (Zhao, Li); Li, XC (Li, Xiangchen); Liu, YL (Liu, Yanlin); Tao, SH (Tao, Shiheng)
**Title:** Training Set Selection for the Prediction of Essential Genes
**Source:** PLOS ONE
**Volume:** 9
**Issue:** 1
**Article Number:** e86805
**DOI:** 10.1371/journal.pone.0086805
**Published:** JAN 22 2014
**Abstract:** Various computational models have been developed to transfer annotations of gene essentiality between organisms. However, despite the increasing number of microorganisms with well-characterized sets of essential genes, selection of appropriate training sets for predicting the essential genes of poorly-studied or newly sequenc organisms remains challenging. In this study, a machine learning approach was applied reciprocally to predict the essential genes in 21 microorganisms. Results showed t training set selection greatly influenced predictive accuracy. We determined four criteria for training set selection: (1) essential genes in the selected training set should be reliable; (2) the growth conditions in which essential genes are defined should be consistent in training and prediction sets; (3) species used as training set should be close related to the target organism; and (4) organisms used as training and prediction sets should exhibit similar phenotypes or lifestyles. We then analyzed the performance of incomplete training set and an integrated training set with multiple organisms. We found that the size of the training set should be at least 10% of the total genes to yield accurate predictions. Additionally, the integrated training sets exhibited remarkable increase in stability and accuracy compared with single sets. Finally, we compared the performance of the integrated training sets with the four criteria and with random selection. The results revealed that a rational selection of training sets based on our crite yields better performance than random selection. Thus, our results provide empirical guidance on training set selection for the identification of essential genes on a genome-wide scale.
**Times Cited in Web of Science Core Collection:** 2
**Times Cited in BIOSIS Citation Index:** 2
**Times Cited in Chinese Science Citation Database:** 0
**Times Cited in Russian Science Citation Index:** 0
**Times Cited in SciELO Citation Index:** 0
**Total Times Cited:** 2
**ISSN:** 1932-6203
**Accession Number:** WOS:000330283100218
**PubMed ID:** 24466248

---

**Record 29 of 38**
**By:** Tang, XW (Tang, Xiwei); Wang, JX (Wang, Jianxin); Zhong, JC (Zhong, Jiancheng); Pan, Y (Pan, Yi)
**Title:** Predicting Essential Proteins Based on Weighted Degree Centrality
**Source:** IEEE-ACM TRANSACTIONS ON COMPUTATIONAL BIOLOGY AND BIOINFORMATICS
**Volume:** 11
**Issue:** 2
**Pages:** 407-418
**DOI:** 10.1109/TCBB.2013.2295318
**Published:** MAR-APR 2014
**Abstract:** Essential proteins are vital for an organism's viability under a variety of conditions. There are many experimental and computational methods developed to ide essential proteins. Computational prediction of essential proteins based on the global protein-protein interaction (PPI) network is severely restricted because of the insufficiency of the PPI data, but fortunately the gene expression profiles help to make up the deficiency. In this work, Pearson correlation coefficient (PCC) is used to br the gap between PPI and gene expression data. Based on PCC and edge clustering coefficient (ECC), a new centrality measure, i.e., the weighted degree centrality (WDC developed to achieve the reliable prediction of essential proteins. WDC is employed to identify essential proteins in the yeast PPI and e-Coli networks in order to estimat performance. For comparison, other prediction technologies are also performed to identify essential proteins. Some evaluation methods are used to analyze the results fro

various prediction approaches. The prediction results and comparative analyses are shown in the paper. Furthermore, the parameter lambda in the method WDC will be analyzed in detail and an optimal lambda value will be found. Based on the optimal lambda value, the differentiation of WDC and another prediction method PeC is discussed. The analyses prove that WDC outperforms other methods including DC, BC, CC, SC, EC, IC, NC, and PeC. At the same time, the analyses also mean that it is effective way to predict essential proteins by means of integrating different data sources.

**Times Cited in Web of Science Core Collection:** 9
**Times Cited in BIOSIS Citation Index:** 7
**Times Cited in Chinese Science Citation Database:** 0
**Times Cited in Russian Science Citation Index:** 0
**Times Cited in SciELO Citation Index:** 0
**Total Times Cited:** 9
**ISSN:** 1545-5963
**eISSN:** 1557-9964
**Accession Number:** WOS:000337147300011
**PubMed ID:** 26355787

---

**Record 30 of 38**
**By:** Lu, Y (Lu, Yao); Deng, JY (Deng, Jingyuan); Rhodes, JC (Rhodes, Judith C.); Lu, H (Lu, Hui); Lu, LJ (Lu, Long Jason)
**Title:** Predicting essential genes for identifying potential drug targets in Aspergillus fumigatus
**Source:** COMPUTATIONAL BIOLOGY AND CHEMISTRY
**Volume:** 50
**Special Issue:** SI
**Pages:** 29-40
**DOI:** 10.1016/j.compbiolchem.2014.01.011
**Published:** JUN 2014
**Abstract:** Background: Aspergillus fumigatus (Af) is a ubiquitous and opportunistic pathogen capable of causing acute, invasive pulmonary disease in susceptible hosts. Despite current therapeutic options, mortality associated with invasive Af infections remains unacceptably high, increasing 357% since 1980. Therefore, there is an urgen need for the development of novel therapeutic strategies, including more efficacious drugs acting on new targets. Thus, as noted in a recent review, "the identification of essential genes in fungi represents a crucial step in the development of new antifungal drugs". Expanding the target space by rapidly identifying new essential genes has t been described as "the most important task of genomics-based target validation".
Results: In previous research, we were the first to show that essential gene annotation can be reliably transferred between distantly related four Prokaryotic species. In thi study, we extend our machine learning approach to the much more complex Eukaryotic fungal species. A compendium of essential genes is predicted in Af by transferrin known essential gene annotations from another filamentous fungus Neurospora crassa. This approach predicts essential genes by integrating diverse types of intrinsic and context-dependent genomic features encoded in microbial genomes. The predicted essential datasets contained 1674 genes. We validated our results by comparing our predictions with known essential genes in Af, comparing our predictions with those predicted by homology mapping, and conducting conditional expressed alleles. We applied several layers of filters and selected a set of potential drug targets from the predicted essential genes. Finally, we have conducted wet lab knockout experiments tc verify our predictions, which further validates the accuracy and wide applicability of the machine learning approach.
Conclusions: The approach presented here significantly extended our ability to predict essential genes beyond orthologs and made it possible to predict an inventory of essential genes in Eukaryotic fungal species, amongst which a preferred subset of suitable drug targets may be selected. By selecting the best new targets, we believe that resultant drugs would exhibit an unparalleled clinical impact against a naive pathogen population. Additional benefits that a compendium of essential genes can provide a important information on cell function and evolutionary biology. Furthermore, mapping essential genes to pathways may also reveal critical check points in the pathogen' metabolism. Finally, this approach is highly reproducible and portable, and can be easily applied to predict essential genes in many more pathogenic microbes, especially those unculturable. (C) 2014 Elsevier Ltd. All rights reserved.
**Times Cited in Web of Science Core Collection:** 0
**Times Cited in BIOSIS Citation Index:** 0
**Times Cited in Chinese Science Citation Database:** 0
**Times Cited in Russian Science Citation Index:** 0
**Times Cited in SciELO Citation Index:** 0
**Total Times Cited:** 0
**ISSN:** 1476-9271
**eISSN:** 1476-928X
**Accession Number:** WOS:000338825600005
**PubMed ID:** 24569026

---

**Record 31 of 38**
**By:** Luo, JW (Luo, Jiawei); Zhang, N (Zhang, Nan)
**Title:** PREDICTION OF ESSENTIAL PROTEINS BASED ON EDGE CLUSTERING COEFFICIENT AND GENE ONTOLOGY INFORMATION
**Source:** JOURNAL OF BIOLOGICAL SYSTEMS
**Volume:** 22
**Issue:** 3
**DOI:** 10.1142/S0218339014500119
**Published:** SEP 2014
**Abstract:** Essential proteins are important for the survival and development of organisms. Lots of centrality algorithms based on network topology have been proposed tc detect essential proteins and achieve good results. However, most of them only focus on the network topology, but ignore the false positive (FP) interactions in protein-protein interaction (PPI) network. In this paper, gene ontology (GO) information is proposed to measure the reliability of the edges in PPI network and we propose a nove algorithm for identifying essential proteins, named EGC algorithm. EGC algorithm integrates topology character of PPI network and GO information. To validate the performance of EGC algorithm, we use EGC and other nine methods (DC, BC, CC, SC, EC, LAC, NC, PEC and CoEWC) to identify the essential proteins in the two different yeast PPI networks: DIP and MIPS. The results show that EGC is better than the other nine methods, which means adding GO information can help in predictin essential proteins.
**Times Cited in Web of Science Core Collection:** 0

**Times Cited in BIOSIS Citation Index:** 0
**Times Cited in Chinese Science Citation Database:** 0
**Times Cited in Russian Science Citation Index:** 0
**Times Cited in SciELO Citation Index:** 0
**Total Times Cited:** 0
**ISSN:** 0218-3390
**eISSN:** 1793-6470
**Accession Number:** WOS:000341496300002

---

**Record 32 of 38**
**By:** Wang, Y (Wang, Yan); Sun, HY (Sun, Huiyan); Du, W (Du, Wei); Blanzieri, E (Blanzieri, Enrico); Viero, G (Viero, Gabriella); Xu, Y (Xu, Ying); Liang, YC (Liang, Yanchun)
**Author Identifiers:**

| Author | ResearcherID Number | ORCID Number |
|---|---|---|
| Du, Wei | J-9804-2015 | 0000-0001-9872-4821 |
| Viero, Gabriella | | 0000-0002-6755-285X |

**Title:** Identification of Essential Proteins Based on Ranking Edge-Weights in Protein-Protein Interaction Networks
**Source:** PLOS ONE
**Volume:** 9
**Issue:** 9
**Article Number:** e108716
**DOI:** 10.1371/journal.pone.0108716
**Published:** SEP 30 2014
**Abstract:** Essential proteins are those that are indispensable to cellular survival and development. Existing methods for essential protein identification generally rely on knock-out experiments and/or the relative density of their interactions (edges) with other proteins in a Protein-Protein Interaction (PPI) network. Here, we present a computational method, called EW, to first rank protein-protein interactions in terms of their Edge Weights, and then identify sub-PPI-networks consisting of only the high ranked edges and predict their proteins as essential proteins. We have applied this method to publicly-available PPI data on Saccharomyces cerevisiae (Yeast) and Escher coli (E. coli) for essential protein identification, and demonstrated that EW achieves better performance than the state-of-the-art methods in terms of the precision-recall a Jackknife measures. The highly-ranked protein-protein interactions by our prediction tend to be biologically significant in both the Yeast and E. coli PPI networks. Furthe analyses on systematically perturbed Yeast and E. coli PPI networks through randomly deleting edges demonstrate that the proposed method is robust and the top-ranked edges tend to be more associated with known essential proteins than the lowly-ranked edges.
**Times Cited in Web of Science Core Collection:** 1
**Times Cited in BIOSIS Citation Index:** 1
**Times Cited in Chinese Science Citation Database:** 0
**Times Cited in Russian Science Citation Index:** 0
**Times Cited in SciELO Citation Index:** 0
**Total Times Cited:** 1
**ISSN:** 1932-6203
**Accession Number:** WOS:000343671700145
**PubMed ID:** 25268881

---

**Record 33 of 38**
**By:** Luo, JW (Luo, Jiawei); Kuang, L (Kuang, Ling)
**Title:** A new method for predicting essential proteins based on dynamic network topology and complex information
**Source:** COMPUTATIONAL BIOLOGY AND CHEMISTRY
**Volume:** 52
**Pages:** 34-42
**DOI:** 10.1016/j.compbiolchem.2014.08.022
**Published:** OCT 2014
**Abstract:** Predicting essential proteins is highly significant because organisms can not survive or develop even if only one of these proteins is missing. Improvements in high-throughput technologies have resulted in a large number of available protein-protein interactions. By taking advantage of these interaction data, researchers have proposed many computational methods to identify essential proteins at the network level. Most of these approaches focus on the topology of a static protein interaction network. However, the protein interaction network changes with time and condition. This important inherent dynamics of the protein interaction network is overlooked b previous methods. In this paper, we introduce a new method named CDLC to predict essential proteins by integrating dynamic local average connectivity and in-degree o proteins in complexes. CDLC is applied to the protein interaction network of Saccharomyces cerevisiae. The results show that CDLC outperforms five other methods (Degree Centrality (DC), Local Average Connectivity-based method (LAC), Sum of ECC (SoECC), PeC and Co-Expression Weighted by Clustering coefficient (CoEWC In particular, CDLC could improve the prediction precision by more than 45% compared with DC methods. CDLC is also compared with the latest algorithm CEPPK, an higher precision is achieved by CDLC. CDLC is available as Supplementary materials. The default settings of active threshold and alpha-parameter are 0.8 and 0.1, respectively. (C) 2014 Elsevier Ltd. All rights reserved.
**Times Cited in Web of Science Core Collection:** 0
**Times Cited in BIOSIS Citation Index:** 0
**Times Cited in Chinese Science Citation Database:** 0
**Times Cited in Russian Science Citation Index:** 0
**Times Cited in SciELO Citation Index:** 0
**Total Times Cited:** 0
**ISSN:** 1476-9271
**eISSN:** 1476-928X

**Accession Number:** WOS:000344427500005
**PubMed ID:** 25179858

---

**Record 34 of 38**
**By:** Zhao, BH (Zhao, Bihai); Wang, JX (Wang, Jianxin); Li, M (Li, Min); Wu, FX (Wu, Fang-xiang); Pan, Y (Pan, Yi)
**Title:** Prediction of Essential Proteins Based on Overlapping Essential Modules
**Source:** IEEE TRANSACTIONS ON NANOBIOSCIENCE
**Volume:** 13
**Issue:** 4
**Pages:** 415-424
**DOI:** 10.1109/TNB.2014.2337912
**Published:** DEC 2014
**Abstract:** Many computational methods have been proposed to identify essential proteins by using the topological features of interactome networks. However, the precis of essential protein discovery still needs to be improved. Researches show that majority of hubs (essential proteins) in the yeast interactome network are essential due to t involvement in essential complex biological modules and hubs can be classified into two categories: date hubs and party hubs. In this study, combining with gene express profiles, we propose a new method to predict essential proteins based on overlapping essential modules, named POEM. In POEM, the original protein interactome netwo partitioned into many overlapping essential modules. The frequencies and weighted degrees of proteins in these modules are employed to decide which categories does a protein belong to? The comparative results show that POEM outperforms the classical centrality measures: Degree Centrality (DC), Information Centrality (IC), Eigenve Centrality (EC), Subgraph Centrality (SC), Betweenness Centrality (BC), Closeness Centrality (CC), Edge Clustering Coefficient Centrality (NC), and two newly propos essential proteins prediction methods: PeC and CoEWC. Experimental results indicate that the precision of predicting essential proteins can be improved by considering t modularity of proteins and integrating gene expression profiles with network topological features.
**Times Cited in Web of Science Core Collection:** 4
**Times Cited in BIOSIS Citation Index:** 3
**Times Cited in Chinese Science Citation Database:** 1
**Times Cited in Russian Science Citation Index:** 0
**Times Cited in SciELO Citation Index:** 0
**Total Times Cited:** 4
**ISSN:** 1536-1241
**eISSN:** 1558-2639
**Accession Number:** WOS:000345906200007
**PubMed ID:** 25122840

---

**Record 35 of 38**
**By:** Xiao, QH (Xiao, Qianghua); Wang, JX (Wang, Jianxin); Peng, XQ (Peng, Xiaoqing); Wu, FX (Wu, Fang-xiang); Pan, Y (Pan, Yi)
**Title:** Identifying essential proteins from active PPI networks constructed with dynamic gene expression
**Source:** BMC GENOMICS
**Volume:** 16
**Article Number:** S1
**DOI:** 10.1186/1471-2164-16-S3-S1
**Supplement:** 3
**Published:** JAN 29 2015
**Abstract:** Essential proteins are vitally important for cellular survival and development, and identifying essential proteins is very meaningful research work in the post-genome era. Rapid increase of available protein-protein interaction (PPI) data has made it possible to detect protein essentiality at the network level. A series of centrality measures have been proposed to discover essential proteins based on the PPI networks. However, the PPI data obtained from large scale, high-throughput experiments generally contain false positives. It is insufficient to use original PPI data to identify essential proteins. How to improve the accuracy, has become the focus c identifying essential proteins. In this paper, we proposed a framework for identifying essential proteins from active PPI networks constructed with dynamic gene expressi Firstly, we process the dynamic gene expression profiles by using time-dependent model and time-independent model. Secondly, we construct an active PPI network base on co-expressed genes. Lastly, we apply six classical centrality measures in the active PPI network. For the purpose of comparison, other prediction methods are also performed to identify essential proteins based on the active PPI network. The experimental results on yeast network show that identifying essential proteins based on the active PPI network can improve the performance of centrality measures considerably in terms of the number of identified essential proteins and identification accuracy. A the same time, the results also indicate that most of essential proteins are active.
**Times Cited in Web of Science Core Collection:** 1
**Times Cited in BIOSIS Citation Index:** 1
**Times Cited in Chinese Science Citation Database:** 0
**Times Cited in Russian Science Citation Index:** 0
**Times Cited in SciELO Citation Index:** 0
**Total Times Cited:** 1
**ISSN:** 1471-2164
**Accession Number:** WOS:000353979000001
**PubMed ID:** 25707432

---

**Record 36 of 38**
**By:** Peng, W (Peng, Wei); Wang, JX (Wang, Jianxin); Cheng, YJ (Cheng, Yingjiao); Lu, Y (Lu, Yu); Wu, FX (Wu, Fangxiang); Pan, Y (Pan, Yi)
**Title:** UDoNC: An Algorithm for Identifying Essential Proteins Based on Protein Domains and Protein-Protein Interaction Networks
**Source:** IEEE-ACM TRANSACTIONS ON COMPUTATIONAL BIOLOGY AND BIOINFORMATICS
**Volume:** 12
**Issue:** 2
**Pages:** 276-288
**DOI:** 10.1109/TCBB.2014.2338317

**Published:** MAR-APR 2015

**Abstract:** Prediction of essential proteins which are crucial to an organism's survival is important for disease analysis and drug design, as well as the understanding of cellular life. The majority of prediction methods infer the possibility of proteins to be essential by using the network topology. However, these methods are limited to the completeness of available protein-protein interaction (PPI) data and depend on the network accuracy. To overcome these limitations, some computational methods have b proposed. However, seldom of them solve this problem by taking consideration of protein domains. In this work, we first analyze the correlation between the essentiality proteins and their domain features based on data of 13 species. We find that the proteins containing more protein domain types which rarely occur in other proteins tend t essential. Accordingly, we propose a new prediction method, named UDoNC, by combining the domain features of proteins with their topological properties in PPI netw In UDoNC, the essentiality of proteins is decided by the number and the frequency of their protein domain types, as well as the essentiality of their adjacent edges measu by edge clustering coefficient. The experimental results on S. cerevisiae data show that UDoNC outperforms other existing methods in terms of area under the curve (AU Additionally, UDoNC can also perform well in predicting essential proteins on data of E. coli.

**Times Cited in Web of Science Core Collection:** 2
**Times Cited in BIOSIS Citation Index:** 2
**Times Cited in Chinese Science Citation Database:** 0
**Times Cited in Russian Science Citation Index:** 0
**Times Cited in SciELO Citation Index:** 0
**Total Times Cited:** 2
**ISSN:** 1545-5963
**eISSN:** 1557-9964
**Accession Number:** WOS:000352788300004
**PubMed ID:** 26357216

---

**Record 37 of 38**
**By:** Li, M (Li, Min); Lu, Y (Lu, Yu); Wang, JX (Wang, Jianxin); Wu, FX (Wu, Fang-Xiang); Pan, Y (Pan, Yi)
**Title:** A Topology Potential-Based Method for Identifying Essential Proteins from PPI Networks
**Source:** IEEE-ACM TRANSACTIONS ON COMPUTATIONAL BIOLOGY AND BIOINFORMATICS
**Volume:** 12
**Issue:** 2
**Pages:** 372-383
**DOI:** 10.1109/TCBB.2014.2361350
**Published:** MAR-APR 2015

**Abstract:** Essential proteins are indispensable for cellular life. It is of great significance to identify essential proteins that can help us understand the minimal requiremen for cellular life and is also very important for drug design. However, identification of essential proteins based on experimental approaches are typically time-consuming a expensive. With the development of high-throughput technology in the post-genomic era, more and more protein-protein interaction data can be obtained, which make it possible to study essential proteins from the network level. There have been a series of computational approaches proposed for predicting essential proteins based on netv topologies. Most of these topology based essential protein discovery methods were to use network centralities. In this paper, we investigate the essential proteins' topolog characters from a completely new perspective. To our knowledge it is the first time that topology potential is used to identify essential proteins from a protein-protein interaction (PPI) network. The basic idea is that each protein in the network can be viewed as a material particle which creates a potential field around itself and the interaction of all proteins forms a topological field over the network. By defining and computing the value of each protein's topology potential, we can obtain a more prec ranking which reflects the importance of proteins from the PPI network. The experimental results show that topology potential-based methods TP and TP-NC outperform traditional topology measures: degree centrality (DC), betweenness centrality (BC), closeness centrality (CC), subgraph centrality (SC), eigenvector centrality (EC), information centrality (IC), and network centrality (NC) for predicting essential proteins. In addition, these centrality measures are improved on their performance for identifying essential proteins in biological network when controlled by topology potential.

**Times Cited in Web of Science Core Collection:** 2
**Times Cited in BIOSIS Citation Index:** 2
**Times Cited in Chinese Science Citation Database:** 0
**Times Cited in Russian Science Citation Index:** 0
**Times Cited in SciELO Citation Index:** 0
**Total Times Cited:** 2
**ISSN:** 1545-5963
**eISSN:** 1557-9964
**Accession Number:** WOS:000352788300012
**PubMed ID:** 26357224

---

**Record 38 of 38**
**By:** Luo, JW (Luo, Jiawei); Qi, Y (Qi, Yi)
**Title:** Identification of Essential Proteins Based on a New Combination of Local Interaction Density and Protein Complexes
**Source:** PLOS ONE
**Volume:** 10
**Issue:** 6
**Article Number:** e0131418
**DOI:** 10.1371/journal.pone.0131418
**Published:** JUN 30 2015

**Abstract:** Background
Computational approaches aided by computer science have been used to predict essential proteins and are faster than expensive, time-consuming, laborious experimental approaches. However, the performance of such approaches is still poor, making practical applications of computational approaches difficult in some fields. Hence, the development of more suitable and efficient computing methods is necessary for identification of essential proteins.
Method
In this paper, we propose a new method for predicting essential proteins in a protein interaction network, local interaction density combined with protein complexes (LID based on statistical analyses of essential proteins and protein complexes. First, we introduce a new local topological centrality, local interaction density (LID), of the yeas

PPI network; second, we discuss a new integration strategy for multiple bioinformatics. The LIDC method was then developed through a combination of LID and protein complex information based on our new integration strategy. The purpose of LIDC is discovery of important features of essential proteins with their neighbors in real prot complexes, thereby improving the efficiency of identification.
Results
Experimental results based on three different PPI(protein-protein interaction) networks of Saccharomyces cerevisiae and Escherichia coli showed that LIDC outperforme classical topological centrality measures and some recent combinational methods. Moreover, when predicting MIPS datasets, the better improvement of performance obtained by LIDC is over all nine reference methods (i.e., DC, BC, NC, LID, PeC, CoEWC, WDC, ION, and UC).
Conclusions
LIDC is more effective for the prediction of essential proteins than other recently developed methods.

**Times Cited in Web of Science Core Collection:** 0
**Times Cited in BIOSIS Citation Index:** 0
**Times Cited in Chinese Science Citation Database:** 0
**Times Cited in Russian Science Citation Index:** 0
**Times Cited in SciELO Citation Index:** 0
**Total Times Cited:** 0
**ISSN:** 1932-6203
**Accession Number:** WOS:000358151300047
**PubMed ID:** 26125187

Close

**Web of Science™**
**Page 1 (Records 1 -- 38)**
[ 1 ]

Pr

© 2015 THOMSON REUTERS TERMS OF USE PRIVACY POLICY FEEDBACK
